# Coarse climate model grids underestimate coastal humid-heat extremes

**DOI:** 10.1126/sciadv.ael4020

**Published:** 2026-10-30

**Authors:** Cosimo E. Carniel, Jonathan D. Wille, Lukas Brunner, Diana Francis, Erich M. Fischer

**Affiliations:** ^1^ Institute for Atmospheric and Climate Sciences, ETH Zürich, Zürich, Switzerland.; ^2^ Institut des Geosciences de l’Environnement, UGA, Saint Martin d’Heres, France.; ^3^ Biogéosciences, CNRS/Université de Bourgogne Europe, Dijon, France.; ^4^ Research Unit Sustainability and Climate Risk, Earth and Society Research Hub (ESRAH), University of Hamburg, Hamburg, Germany.; ^5^ ENGEOS Lab, Department of Earth Sciences, Khalifa University, Abu Dhabi, United Arab Emirates.

## Abstract

Humid-heat extremes reach intensities close to the limit of human survivability around the Arabian Peninsula. Here, we analyze fully coupled kilometer-scale Icosahedral Nonhydrostatic (ICON) and Integrated Forecasting System–FinitevolumE Sea ice-Ocean Model (IFS-FESOM) simulations from the nextGEMS project, with original resolutions of around 9 kilometers, and compare them with station observations, ERA5, and CMIP6 projections. IFS-FESOM reproduces well the observed upper-tail wet-bulb temperature (TW) and lies near the high end of the CMIP6 distribution over coastal regions, while ICON simulates weaker extremes. Spatial smoothing suppresses localized coastal maxima, reducing daily-maximum TW extremes by up to 8°C and lowering exceedance frequencies across multiple thresholds. Native resolution fields also show stronger mid-century increases in exposure under SSP3-7.0 than their coarse-gridded counterparts. These results suggest that conventional coarse resolution projections may underestimate localized coastal humid-heat risk, although the magnitude of this effect remains model dependent.

## INTRODUCTION

Extreme dry heat and humid-heat events are becoming increasingly frequent and severe, leading to excess mortality, reducing labor productivity, causing stress on ecosystems and exacerbating socio-economic inequalities (*1*, *2*). In regions where both temperature and humidity are high (*3*–*5*), evaporative cooling becomes ineffective, critically impairing the body’s ability to regulate internal temperature.

The Arabian Peninsula [AP; (*6*, *7*)] while generally being arid, is among the regions most affected by humid-heat extremes (*8*). Exceptionally high sea surface temperatures (SSTs), which can exceed 37°C (*9*), together with the inland transport of warm, moist air and intense continental heating, produce wet-bulb temperature (TW) values approaching physiological limits even under present-day climate conditions (*10*). Several studies project a strong increase in TW extremes in the AP by the end of the century (*7*), including frequent exceedance of the commonly used, although likely conservative (*11*), 35°C survivability threshold. These risks are amplified by the region’s above-average warming rate (*4*, *12*, *13*), of about 0.6°C per decade from 1978 to 2019 (*14*, *15*), compared with a global mean increase of about 0.17°C per decade (*16*).

Despite their importance, projections of humid heat remain less investigated than those for dry heat. Global climate models (GCMs) have provided valuable insights into large-scale warming, but their coarse resolution (50 to 250 km) limits the representation of key local processes such as coastal dynamics, mesoscale circulation, air-sea feedbacks, and the triggering and intensity of moist convection (*17*). This potentially leads to underestimations of both the intensity and coastal variability of TW extremes.

Kilometer-scale models offer a new avenue to address this challenge. With a typical grid resolution of 5 to 10 km, these simulations capture fine-scale interactions between land, sea, and atmosphere (*18*), more realistically representing sea-breeze dynamics and orographic circulation, which strongly influence coastal TW extremes.

Here, we analyze fully coupled kilometer-scale simulations from the Icosahedral Nonhydrostatic (ICON) and Integrated Forecasting System–FinitevolumE Sea ice-Ocean Model (IFS-FESOM) Earth system models produced within the nextGEMS project (*19*). We use these simulations to assess how high resolution affects the intensity, spatial distribution, and projected changes of TW extremes. Model performance is evaluated against ERA5 reanalysis and station observations from GHCNh (*20*, *21*), while CMIP6 simulations are used as a coarse resolution benchmark to place the kilometer-scale results in the context of conventional global climate projections.

## RESULTS

### Humid-heat extreme characteristics against observations

Humid-heat extremes can reach very different intensities between coastal regions, inland deserts, and elevated terrain because they depend on both temperature and humidity. Compared with station observations from the Global Historical Climatology Network hourly dataset (GHCNh), ERA5 tends to underestimate the upper tail of TW values, consistent with earlier studies (*10*).

Much of the region shows an underestimation of station-based TW99 in ERA5, with deviations of −1° to −3°C at most coastal and inland stations (Fig. 1A). This is observable especially along the eastern and southern coasts of the Persian Gulf and parts of the Red Sea coastline, where differences locally exceed −4°C. Although station records may also include measurement uncertainties, these discrepancies may partly stem from the coarser ERA5 grid and mismatches between station and grid cell elevation or coastal exposure.

**Fig. 1.**
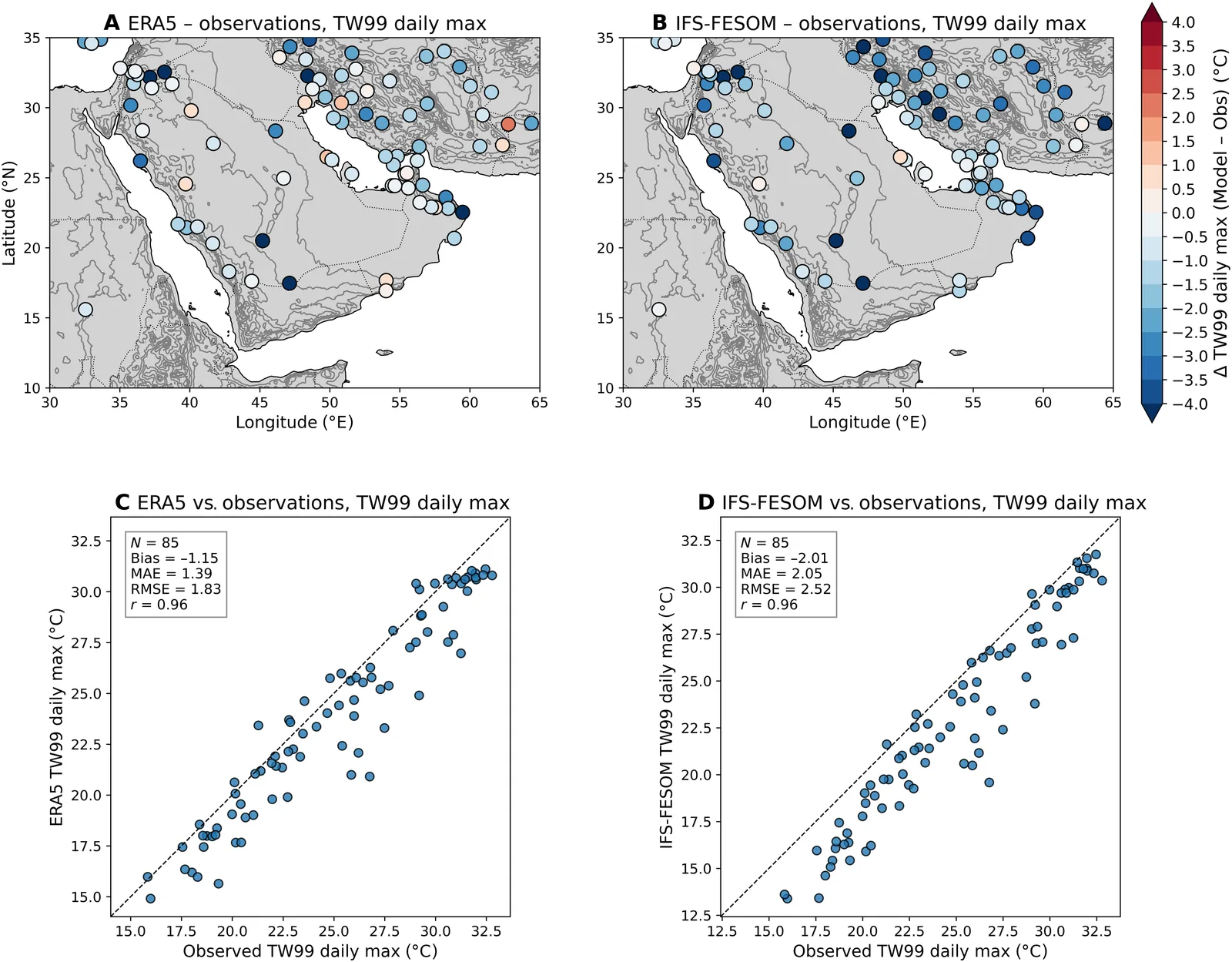
Evaluation of local humid-heat extremes in ERA5 and IFS-FESOM against station observations. Comparison of the 99th percentile of daily maximum TW (TW99) from GHCNh station observations with ERA5 reanalysis and the IFS-FESOM historical simulation over the Arabian Peninsula. (**A**) and (**B**) show the spatial distribution of biases at the nearest grid point, calculated as model/reanalysis minus observations. Positive values indicate overestimation and negative values indicate underestimation relative to station observations. (**C**) and (**D**) show the corresponding station-by-station scatter plots for ERA5 and IFS-FESOM, respectively, with the dashed line indicating the 1:1 relationship. The inset boxes report the number of stations, mean bias, mean absolute error (MAE), root-mean-square error (RMSE), and spatial correlation coefficient. All data span the period 1990 to 2019. Only stations with at least 10 years of records after 1990 are included, yielding 85 stations. Coastlines and national boundaries are from Natural Earth (public domain).

The station-by-station comparison further quantifies these differences (Fig. 1, C and D). Both ERA5 and IFS-FESOM reproduce the spatial variability of observed TW99 well, with most stations following the one-to-one relationship. However, the scatter plots also confirm that both datasets retain a systematic low bias relative to observations.

The free-running coupled IFS-FESOM simulation, run at higher resolution than ERA5, exhibits biases close to zero at many coastal stations along the southeastern Persian Gulf, particularly near Dubai and Abu Dhabi (Fig. 1B). In other locations, TW99 is overestimated by no more than +1° to +2°C, especially around Qatar and Bahrain, as well as along the southern Red Sea coast. ICON does not include a comparable historical simulation, so it was not evaluated against ERA5 and observations.

Over elevated terrain, such as the Asir and Hijaz Mountains, both ERA5 and IFS-FESOM exhibit substantial discrepancies relative to station data. Topographic complexity likely contributes to mismatches between the elevation of the stations and model grid cells (*22*). Overall, the comparison indicates that IFS-FESOM represents the spatial variability of station-based TW99 well, while both datasets retain a systematic low bias relative to observations.

### Higher coastal extremes at higher resolution

A central question is whether kilometer-scale models can capture humid-heat extremes that would be potentially missed at coarser resolutions. Because no directly comparable coarser resolution simulation is available within nextGEMS, we address this question here by coarse gridding the high-resolution simulation for individual variables to typical CMIP model resolutions (*23*). Both ICON and IFS-FESOM are discretized on a HEALPix grid (*24*), which allows consistent aggregation between different “zoom” levels, corresponding here to horizontal resolutions of 13 km (zoom9), 51 km (zoom7), and 204 km (zoom5).

To evaluate the resolution effects, we applied two complementary strategies: (i) the MEAN method, where the output fields of the variables needed to calculate TW were spatially averaged to coarser grids before recomputing TW, and (ii) the MAX method, where TW fields were aggregated directly while retaining the local maxima from the finest grid as representative of coarser resolutions (see “Materials and Methods”). The former approximates the world “as seen” by a coarse-resolution model, while the latter underlines the local extremes potentially captured only at kilometer scales, revealing what traditional models could miss. Together, they provide a conservative estimate of the added value of high resolution in capturing the local and potentially highly impact-relevant TW maxima because they still benefit from the resolved processes in the high resolution model. As depicted in Fig. 2 (A and D), coastal regions consistently emerge as the primary hotspots across the AP, with the Persian Gulf and Red Sea standing out as the most critical regions in both models and ERA5. In the Red Sea, sharp orographic gradients created by the Asir and Sarawat Mountains amplify extremes along narrow coastal strips, while the arid interior of Saudi Arabia and Oman remains far less affected. Composite analysis of extreme events (fig. S1) also reveals how the thermal, moisture, and wind components combine to generate these hotspots: High temperatures exceeding 30°C interact with elevated relative humidity likely advected from nearby seas, producing conditions where evaporative cooling is severely limited (*7*). Coastal sea breezes plausibly reinforce this pattern, with weak onshore winds concentrating humidity near shorelines, while orographic barriers confine moist air masses. In the Red Sea, convergence of desert air and maritime inflow intensifies humid heat along the coast, while in the Persian Gulf, localized convergence north of the basin sustains the highest extremes.

**Fig. 2.**
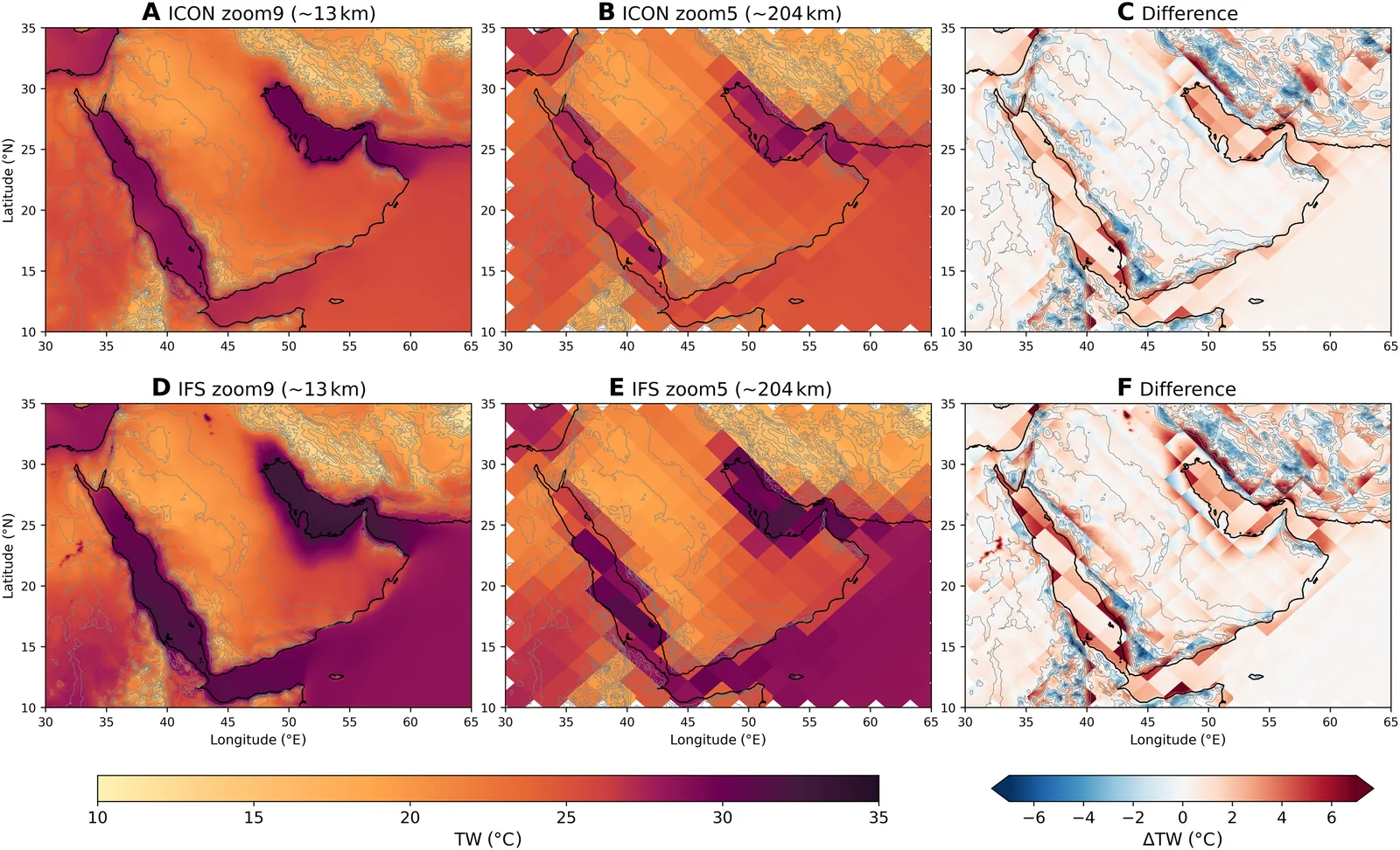
Coastal humid-heat maxima smoothed out at coarser resolution. 99th percentile of daily maximum TW in degrees Celsius from ICON (top row) and IFS-FESOM (bottom row) at different spatial resolutions: (**A** and **D**) zoom9 (∼13 km), (**B** and **E**) zoom5 (∼204 km), obtained through TW recomputation after aggregation of the input variables with the MEAN method, and (**C** and **F**) the difference between zoom9 and zoom5. For the difference maps, the zoom5 fields are first expanded back onto the zoom9 grid by assigning each coarse-cell value to its corresponding native resolution cells; the plotted difference is zoom9 minus expanded zoom5. Coastlines and national boundaries are from Natural Earth (public domain).

Comparing the two resolutions reveals that, in their native grid, both models produce sharper coastal gradients and more localized maxima than at coarser resolutions (Fig. 2). Aggregation smooths these patterns, broadening peaks and hiding finer-scale variability, particularly along the southern Red Sea and eastern Persian Gulf. To compute the difference maps, the zoom5 fields were first expanded back onto the zoom9 grid by assigning each coarse-cell value to its corresponding native resolution cells; the plotted difference is then zoom9 minus expanded zoom5. These maps (Fig. 2, C and F) reveal a systematic underestimation of extreme intensity at coarser scales, with widespread deviations of 2° to 4°C, strongly tied to coastal topography, and local differences exceeding 6°C in ICON and 7°C in IFS-FESOM. Along mountain ranges, the coarse resolution also often shows higher maxima, most likely because it does not capture elevation gradients.

Yet, it remains unclear whether this misrepresentation of local maxima reflects a genuine resolution effect or is simply related to the averaging procedure. To disentangle the two, we next applied the MAX method and compared its results against values recomputed from averaged inputs (Fig. 3).

**Fig. 3.**
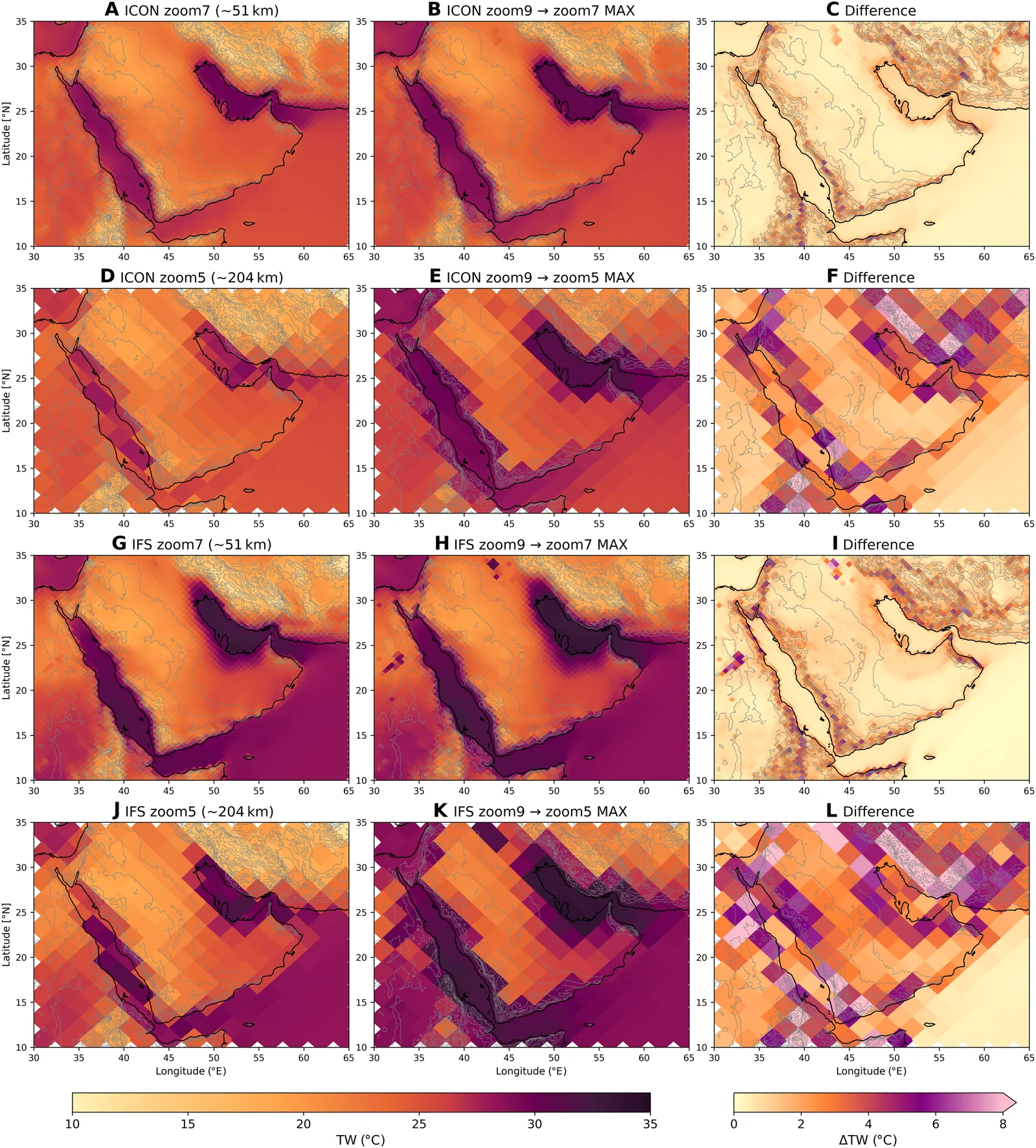
More intense humid-heat extreme maxima at kilometer-scale resolution. 99th percentile of daily maximum TW from ICON and IFS-FESOM. Results are shown for grid aggregations from zoom9 to zoom7 (first and third rows) and from zoom9 to zoom5 (second and fourth rows). For each model, the left column (**A**, **D**, **G**, and **J**) shows TW calculated after aggregating the input variables using the MEAN method; the middle column (**B**, **E**, **H**, and **K**) shows TW aggregated using the MAX method, i.e., retaining maxima from the native resolution; the right column (**C**, **F**, **I**, and **L**) shows the differences between the two aggregation approaches (middle minus left column). Coastlines and national boundaries are from Natural Earth (public domain).

Both models exhibit a systematic underestimation of maxima at coarser scales. When moving from zoom9 to zoom7, deviations of 1° to 2°C are common along immediate coastlines and rise to 3° to 4°C in orographically complex regions such as the Hejaz and Zagros ranges. The contrast becomes more notable at zoom5, a resolution still common in CMIP6 models, where the loss of fine-scale information produces biases of up to 7°C in ICON and more than 8°C in IFS-FESOM. These deviations are consistently clustered in coastal and mountainous regions, where sea-breeze circulations and topography-related processes likely play a more decisive role in shaping elevated humid-heat instances. This highlights the first key effect of higher resolution: capturing stronger intensities of TW extremes. These are also regions with typically high population density, where even 1° to 2°C is highly impact relevant, let alone an underestimation of more than 5°C.

This coarse-grid recomputation framework does not consist in an independent low-resolution climate-model experiment. Instead, it tests how the diagnosis of TW changes when the underlying temperature, humidity, and pressure fields are available only as coarser-grid mean states, and contrasts this with the native-resolution maxima that are lost within each coarse cell. We therefore complement this experiment with a direct benchmark against raw CMIP6 models.

### Kilometer-scale simulations in the context of raw CMIP6 models

To place the kilometer-scale simulations in the context of actual coarse resolution climate models, we compared ICON and IFS-FESOM with raw CMIP6 models over the same region, period, and season (Fig. 4). For this comparison, we used one realization from each CMIP6 model providing the TW metric under SSP3-7.0. Because the available CMIP6 product computes TW from daily maximum near-surface temperature, daily mean near-surface specific humidity, and daily mean surface pressure, all boxplot comparisons involving CMIP6 use this same daily input convention. We therefore recomputed ICON and IFS-FESOM TW in this “CMIP6-way” before comparing them with the raw CMIP6 ensemble. The comparison was restricted to JJA 2024 to 2049 and to the three analysis masks: the AP domain, the Red Sea coastal region, and the Persian Gulf coastal region (see “Materials and Methods”). For each model and region, we computed the grid-cell TW99 and summarized its spatial distribution using the median, 95th percentile, and maximum.

**Fig. 4.**
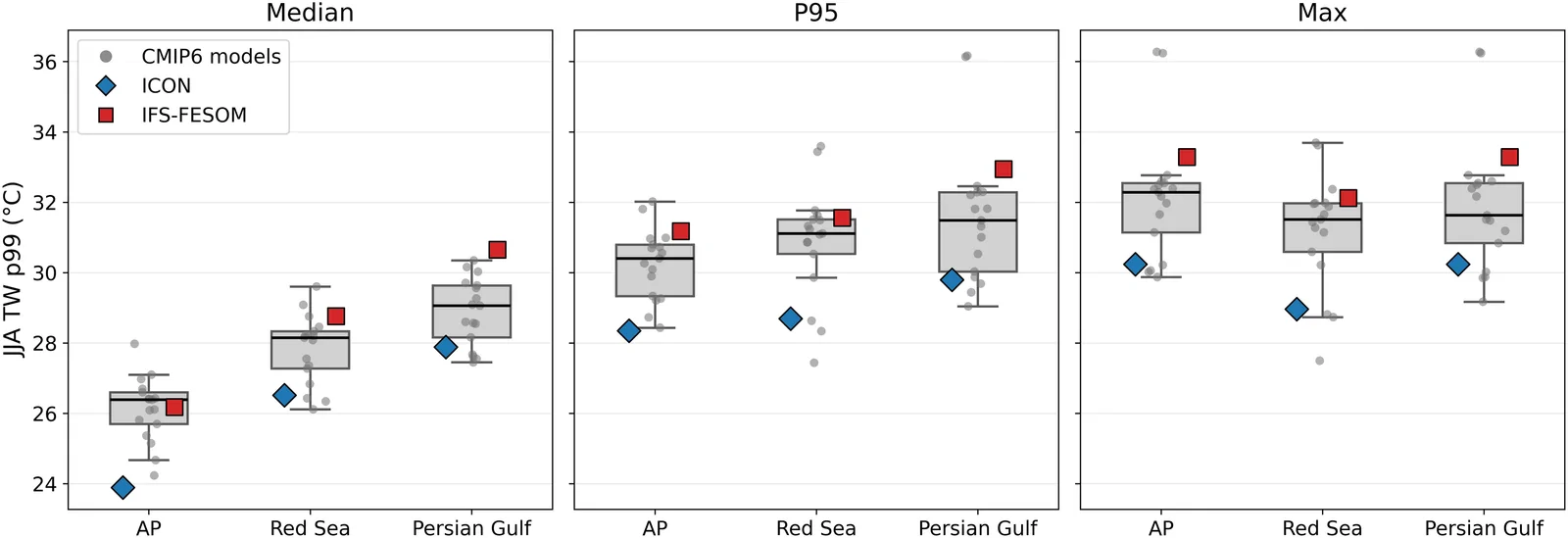
Raw CMIP6 benchmark of JJA TW extremes against nextGEMS ICON and IFS-FESOM. For consistency with the CMIP6 heat-stress product, TW in this figure is computed using the CMIP6 daily input convention for both CMIP6 and nextGEMS. For each model, TW99 is computed at each grid cell over 2024 to 2049, and the panels show the spatial median, spatial 95th percentile, and spatial maximum within the Arabian Peninsula, Red Sea, and Persian Gulf masks. Gray dots show individual CMIP6 models. Boxes show the interquartile range (25th to 75th percentile) across CMIP6 models, with the horizontal line indicating the CMIP6 median; whiskers extend to the most extreme values within 1.5 times the interquartile range. Blue diamonds and red squares show ICON and IFS-FESOM, respectively.

The CMIP6 ensemble spans a broad range of values, indicating that differences in model physics and mean state strongly affect simulated humid heat. The kilometer-scale simulations do not sit uniformly above the CMIP6 range. ICON generally lies in the lower part of the CMIP6 distribution, whereas IFS-FESOM reaches substantially higher values, particularly for the coastal upper-tail metrics. This contrast is especially relevant in light of the station comparison above: IFS-FESOM retains a modest cold bias relative to observations for TW99; its position near or above the upper part of the CMIP6 distribution therefore suggests that many CMIP6 models may underestimate localized coastal upper-tail humid heat, especially in the Red Sea and Persian Gulf regions.

The comparison also highlights substantial differences between the two nextGEMS models. ICON produces weaker humid-heat extremes than IFS-FESOM and often lies close to the lower or middle part of the CMIP6 distribution, especially in the coastal upper tail. This is consistent with the broader nextGEMS evaluation, which reports a colder global near-surface climate in ICON-C4 than in IFS-FESOM and HadCRUT during the early production period, as well as an initial cooling in ICON in contrast to IFS-FESOM (*19*).

The CMIP6 comparison therefore supports a more nuanced conclusion. Kilometer-scale resolution can sharpen localized coastal humid-heat extremes, but the regional baseline of those extremes remains strongly model dependent. In particular, IFS-FESOM reaches the high end of the CMIP6 distribution despite retaining a low bias relative to station observations, suggesting that many coarse-resolution models may miss part of the localized coastal upper tail. Some CMIP6 models nevertheless produce TW extremes higher than IFS-FESOM; if such models were run at higher resolution, these extremes could plausibly become even larger, provided that the model-physics changes required for higher resolution did not substantially alter the regional mean state. At the same time, the weaker ICON response shows that resolution alone is not sufficient: The simulated magnitude of humid-heat extremes also depends strongly on the model formulation and baseline state.

### More frequent events at high resolution

To examine the frequency of occurrence of coastal extreme TW values, we first evaluated exceedances of the empirical physiological threshold proposed by Vecellio *et al.* (*25*), hereafter the Vecellio threshold. For the warm-humid regime, this threshold corresponds to TW ≈ 30.6°C. Because the threshold is regime dependent, we tested whether the strongest simulated TW events fall within the warm-humid regime for which this value is appropriate. The upper-tail events in the coastal masks are predominantly associated with this regime.

Because exceedances of the Vecellio threshold are nearly absent in ICON, the spatial exceedance maps for this threshold are shown only for IFS-FESOM. Using this threshold, IFS-FESOM at native resolution shows narrow exceedance bands tightly following the coasts of the two water bodies, especially around the Persian Gulf, where the average number of exceedances reaches around 80 days per year (Fig. 5). As resolution is coarsened, these fine-scale coastal structures are progressively smoothed. At zoom7, the main hotspots remain visible but with lower frequencies, while at zoom5, much of the spatial detail disappears. In the Persian Gulf zoomed-in panels, the high-frequency coastal band is strongly reduced, showing that coarse resolution underestimates the intensity of humid-heat extremes and, as a consequence, the period during which populations are exposed to potentially critical conditions. A similar coastal footprint is also visible for the fixed 28°C diagnostic threshold, particularly along the southern Red Sea, where the native resolution fields show a narrow band of frequent exceedances that is progressively smoothed at zoom7 and largely lost at zoom5 (fig. S2).

**Fig. 5.**
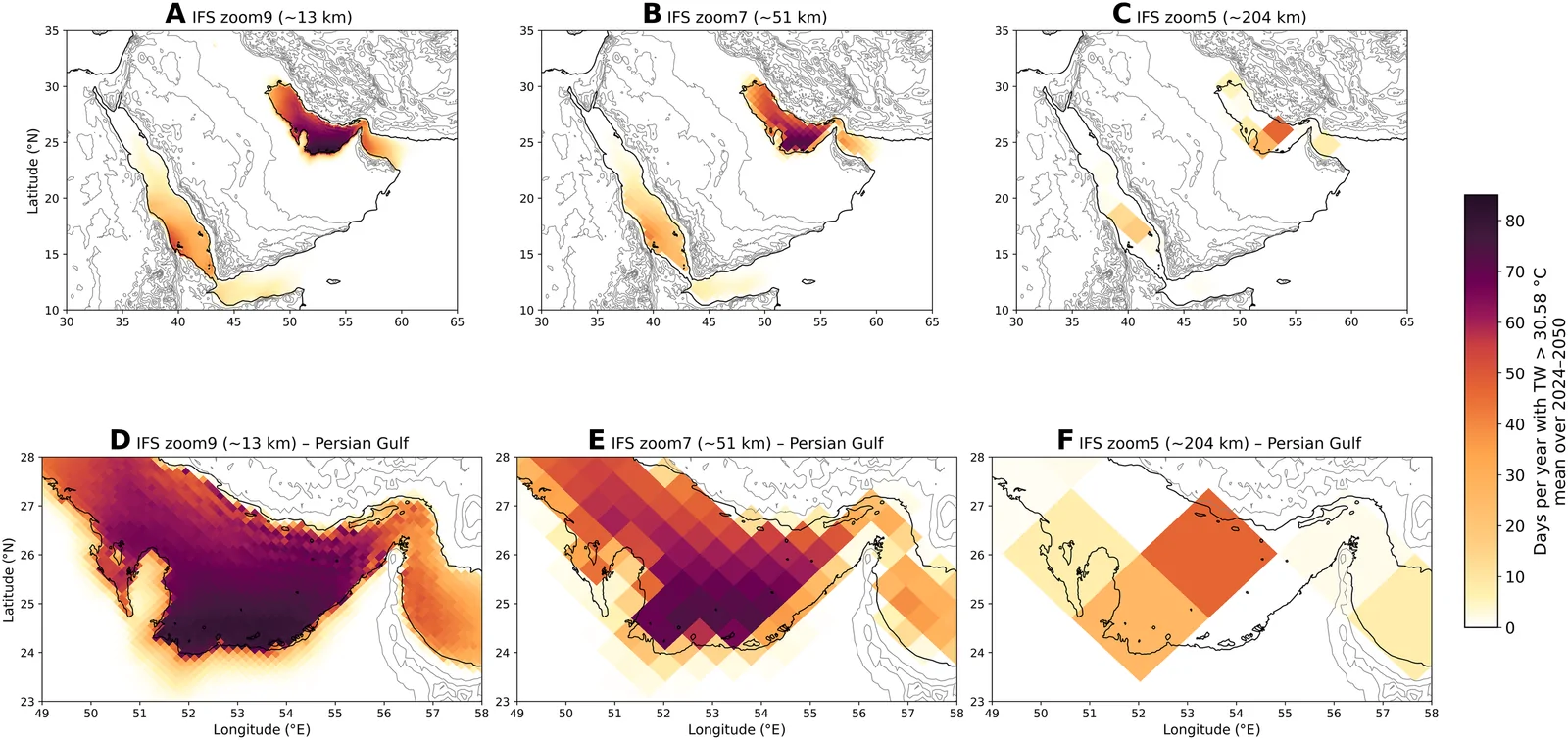
Frequency and persistence of humid-heat extremes strongly underestimated at coarser resolution. Average annual exceedances of the Vecellio 30.6°C TW threshold during 2024 to 2049. Panels show the average number of days per year exceeding the threshold in IFS-FESOM (**A** to **C**) at zoom9 (left), zoom7 (middle), and zoom5 (right) resolutions. Exceedances in the coarser grids (zoom7 and zoom5) are derived by aggregating the input variables for the computation of TW using the MEAN method; (**D**) to (**F**) provide a zoomed-in view of the Persian Gulf coast. Coastlines and national boundaries are from Natural Earth (public domain).

We then tested whether this resolution-related underestimation depends on the specific threshold choice by comparing mean exceedance frequencies for three complementary threshold definitions: a fixed 28°C threshold, the Vecellio threshold, and a percentile-based threshold (Fig. 6). The percentile-based threshold is included to provide a resolution-specific diagnostic that is less dependent on the choice of an absolute TW value. For ICON and IFS-FESOM, we first diagnose the local TW99 from the coarse zoom5 representation and then evaluate how often the native zoom9 grid cells exceed this coarse-grid extreme threshold. This asks how much localized kilometer-scale exceedance activity is hidden within a coarse grid cell. It should therefore not be interpreted as an independent low-resolution model experiment, but as a diagnostic of the exceedances that are lost when coastal extremes are spatially smoothed.

**Fig. 6.**
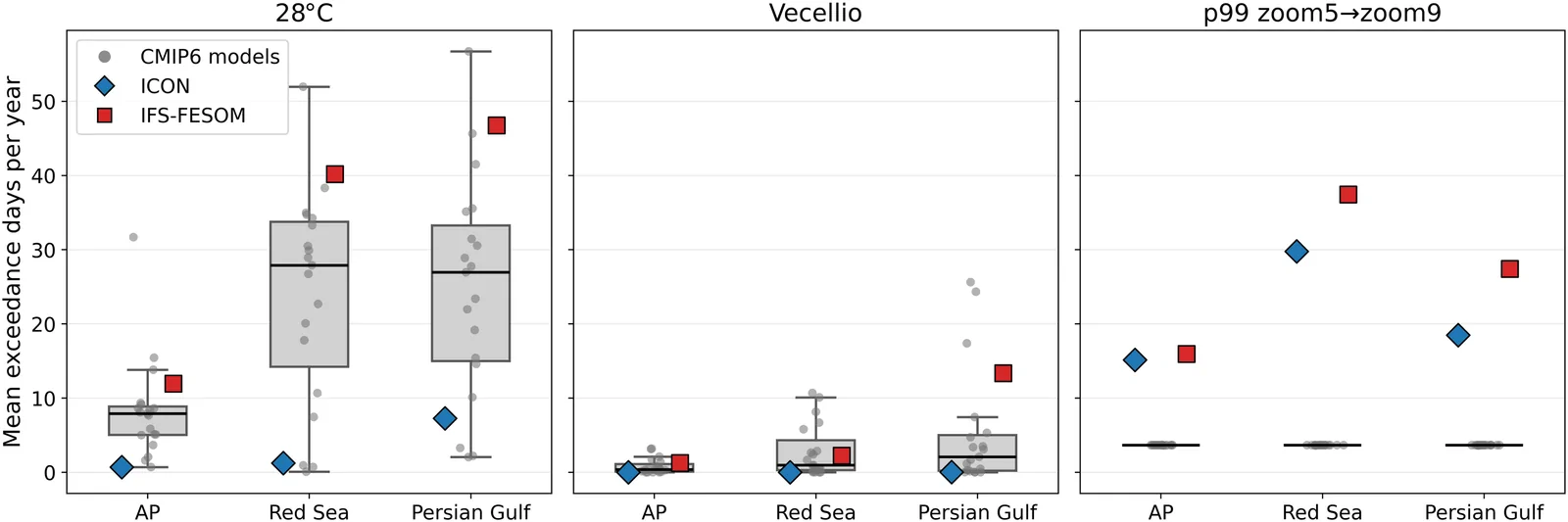
Mean TW exceedance frequency over 2024 to 2049. Values show the average number of exceedance days per year within the Arabian Peninsula, Red Sea, and Persian Gulf masks. The three panels use different threshold definitions: a fixed TW > 28°C threshold, the Vecellio physiological threshold (TW > 30.6°C), and a model-specific cell-wise 99th-percentile threshold. In the last panel, CMIP6 models are evaluated against their own local TW99 threshold, while ICON and IFS-FESOM are evaluated against the TW99 threshold diagnosed from their zoom5 representation and expanded back to zoom9. Gray dots show individual raw CMIP6 models. Boxes show the interquartile range (25th to 75th percentile) across CMIP6 models, with the horizontal line indicating the CMIP6 median; whiskers extend to the most extreme values within 1.5 times the interquartile range. Blue diamonds and red squares show ICON and IFS-FESOM, respectively. All values are based on the CMIP6 daily input convention for TW.

Across the different thresholds, IFS-FESOM frequently lies above the CMIP6 interquartile range, especially in the Red Sea and Persian Gulf regions, indicating more frequent localized humid-heat exceedances than in the central range of coarse-resolution models. This is particularly clear for the zoom5 percentile threshold, where native kilometer-scale grid cells frequently exceed the extreme threshold diagnosed from their coarser representation. ICON, in contrast, generally remains lower than IFS-FESOM and closer to the lower part of the CMIP6 distribution, consistent with its colder mean-state conditions discussed above. The same qualitative result therefore emerges across threshold definitions: Exceedance frequencies are larger when localized kilometer-scale coastal extremes are retained, and the loss of exceedance frequency at coarse resolution is not an artifact of a single threshold choice.

### Enhanced climate change signal

The resolution also affects the climate-change signal in exceedance frequency between the near-term (2024 to 2036) and mid-century (2037 to 2049) periods under SSP3-7.0. Despite the short simulation period, resulting from the high computational cost of the setup, IFS-FESOM still projects an increase in the number of days exceeding the Vecellio threshold, especially along coastal and maritime regions (Fig. 7). This increase is strongest and most spatially coherent at native resolution, where the Persian Gulf and southern Red Sea show widespread positive changes. At zoom7, the main pattern remains but is weakened, while at zoom5, much of the coastal structure is smoothed out and the strongest local signals are substantially reduced.

**Fig. 7.**
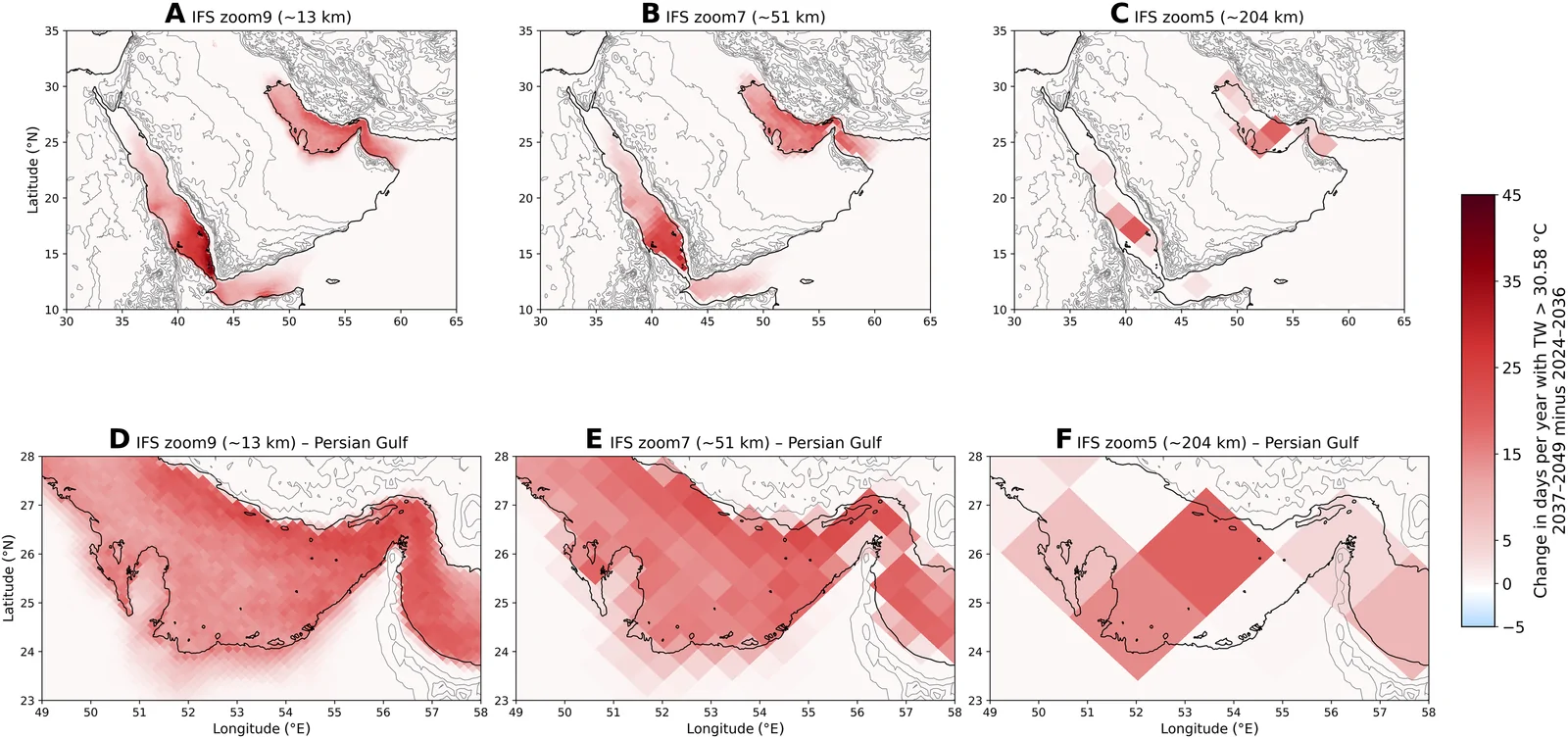
Stronger change in humid-heat exceedance frequency at kilometer-scale resolution. Difference in the average number of days per year exceeding the Vecellio threshold between the periods 2037 to 2049 and 2024 to 2036 across different resolutions of IFS-FESOM (**A** to **C**) at zoom9 (left), zoom7 (middle), and zoom5 (right) resolutions. Exceedances in the coarser grids (zoom7 and zoom5) are derived by aggregating the input variables for the computation of TW using the MEAN method. (**D**) to (**F**) provide a zoomed-in view of the Persian Gulf coast. Coastlines and national boundaries are from Natural Earth (public domain).

This indicates that coarse gridding not only reduces the mean exceedance frequency, but also weakens the diagnosed climate-change signal. Given a higher absolute exceedance frequency at native resolution, the same overall warming and shift in the TW distribution can translate into a larger increase in threshold exceedances (*26*). While this follows from simple statistical considerations, it has important implications for impact-oriented humid-heat indices: Coarse resolution output may underestimate both the present-day exposure and its projected intensification. This behavior is again mirrored for changes in exceedances of the fixed 28°C threshold, with the strongest increases concentrated along the southern Red Sea and Persian Gulf at native resolution, while the coastal signal becomes weaker and less spatially coherent after coarse gridding (fig. S3).

The same threshold framework was then applied to the climate change signal, comparing changes in mean exceedance frequency between 2024 to 2036 and 2037 to 2049 across CMIP6, ICON, and IFS-FESOM (Fig. 8).

**Fig. 8.**
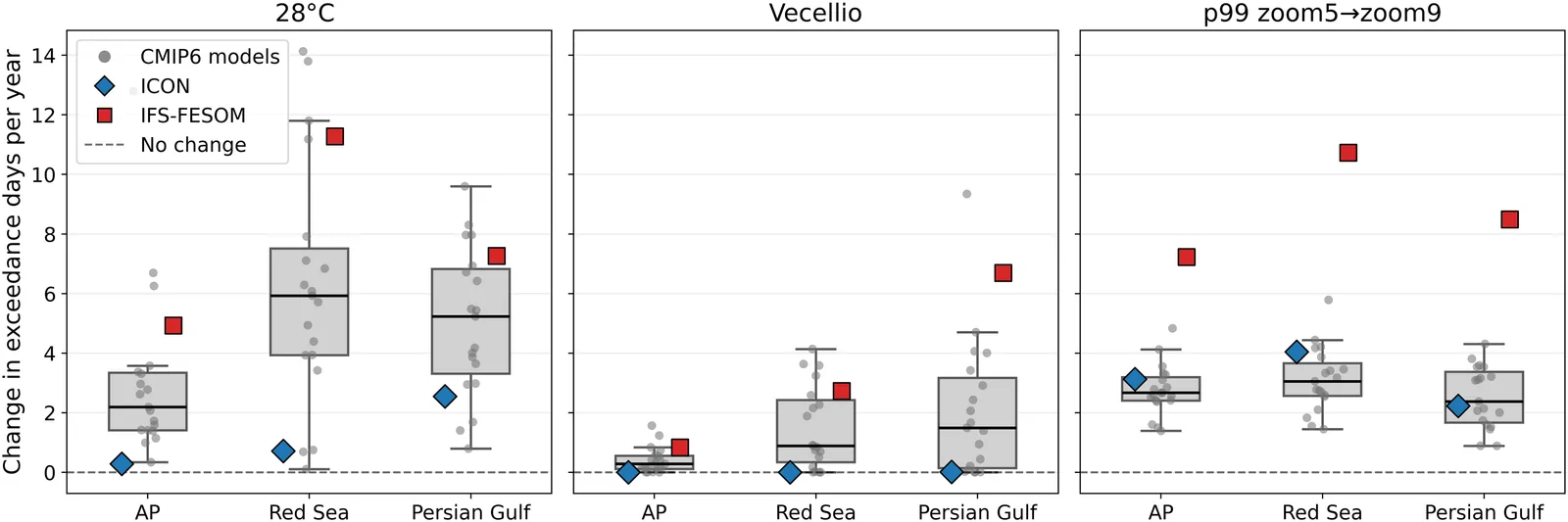
Change in TW exceedance frequency between 2037 to 2049 and 2024 to 2036. Values show the difference in the average number of exceedance days per year within the Arabian Peninsula, Red Sea, and Persian Gulf masks. The three panels use different threshold definitions: a fixed TW > 28°C threshold, the Vecellio physiological threshold (30.6°C), and a 99th-percentile threshold. In the last panel, CMIP6 models are evaluated against their own local TW99 threshold, while ICON and IFS-FESOM are evaluated against the TW99 threshold diagnosed from their zoom5 representation and expanded back to zoom9. Gray dots show individual raw CMIP6 models, boxes show their intermodel distribution, and blue diamonds and red squares show ICON and IFS-FESOM, respectively. All values in this boxplot are based on the CMIP6 daily-input convention for TW.

The increase is positive for almost all models and regions, confirming a general intensification of humid-heat exceedances by mid-century. However, the magnitude of this increase differs strongly across models and thresholds. IFS-FESOM shows the largest changes, particularly in the Red Sea and Persian Gulf regions, where it often exceeds the CMIP6 interquartile range and indicates a stronger localized coastal intensification than most coarse resolution models. This is especially clear for the fixed 28°C and zoom5 percentile thresholds, where the coastal exceedance frequencies are already high in the reference period and therefore respond strongly to a further shift in the TW distribution. ICON consistently shows weaker changes and generally remains closer to the lower part of the CMIP6 distribution.

Overall, the threshold-based comparison shows that the resolution dependence identified for mean exceedance frequency also affects the projected climate change signal and suggests how retaining kilometer-scale coastal extremes leads to a stronger diagnosed increase in humid-heat exposure, whereas coarse spatial smoothing reduces the apparent intensification.

## DISCUSSION

In this study, we show that changing the resolution of model output from about 200 km to about 10 km substantially alters the representation of humid-heat extremes in the AP. Kilometer-scale models capture sharper coastal gradients, higher local maxima, and more frequent TW exceedances than coarse-gridded output, particularly in coastal and topographically complex regions. The direct comparison with raw CMIP6 models adds an important nuance to this result. The kilometer-scale simulations do not uniformly sit above the CMIP6 range: IFS-FESOM reaches and surpasses the high end of the CMIP6 distribution for coastal upper-tail humid heat, whereas ICON often lies in the lower part of the distribution. Because IFS-FESOM still retains a low TW bias relative to station observations, this suggests that many coarser resolution models may miss part of the localized coastal upper tail. At the same time, the weaker ICON response shows that high resolution alone is not sufficient; model formulation and mean-state biases remain central.

The controlled coarse-gridding experiments clarify why resolution matters. Local maxima are up to 7° to 8°C lower after MEAN aggregation to CMIP-like scales than when the high resolution maxima are retained. Exceedance frequencies are also reduced, a result that is robust across several different thresholds, while the projected climate change signal is similarly weakened. While both models project increasing humid-heat exceedances under SSP3-7.0, in agreement with previous studies (*27*, *28*), the diagnosed increase is strongest when kilometer-scale coastal extremes are retained. This behavior can be partly understood statistically: When the reference period exceedance frequency is already higher at native resolution (as is the case for IFS-FESOM), a further shift in the TW distribution produces a larger increase in threshold exceedances. Given the nonlinear relationship between TW and human heat stress, such differences can be highly relevant (*29*).

The physical interpretation is consistent with the regional setting of the AP. Extreme TW depends on the co-occurrence of high temperature and high humidity, and the strongest values are concentrated where land-sea contrasts, shallow warm seas, sea-breeze circulations, and orographic confinement interact. A complementary decomposition (see figs. S4 and S5) suggests that nearly two-thirds of the summer TW trend arises from increasing specific humidity, with about one-third directly linked to temperature, consistent with earlier studies (*30*–*33*). Higher resolution may therefore be especially important where moisture-related processes and coastal confinement amplify humid heat along narrow coastal zones (*7*).

This could have implications beyond the AP. Similar combinations of coastal exposure, complex topography, strong land-sea gradients, and dense populations occur in other regions, including the Mediterranean Basin, Central North America (*17*), and the coastal regions of India, Bangladesh, and China. TWs around the Mediterranean do not typically reach the same absolute values as in the AP. However, the region shows increasing TW trends (*3*), and its coastlines share several features that can make coarse resolution projections miss localized humid-heat exposure.

Nevertheless, several limitations remain. Our analysis is based on two of the first multidecadal fully coupled kilometer-scale model simulations, which are among the longest currently available, but they are still relatively short and are sensitive to internal variability. A clean comparison would be difficult, because changing resolution would also require changes in parameterizations and model formulation. We therefore approximate the effect of lower resolution by coarse gridding the model output with the MEAN and MAX methods. This should not be interpreted as an independent low-resolution climate model experiment, but rather as a diagnostic of how much local information is lost when the same simulated atmosphere is viewed at coarser resolution. In this sense, the coarse-gridded results are likely conservative, because they still benefit from small-scale processes represented in the original kilometer-scale simulations.

The comparison with CMIP6 also has to be interpreted carefully. CMIP6 models differ from nextGEMS not only in resolution, but also in model physics, parameterizations, and mean-state biases. The contrast between ICON and IFS-FESOM shows that these factors can strongly affect humid-heat extremes, even at similar nominal resolution. In addition, in situ station data in the region remain sparse (*34*, *35*), limiting the observational constraints on upper-tail humid heat. Understanding the root causes of the intermodel differences is therefore an important next step. Future work should use multimember ensembles of kilometer-scale models to better separate forced changes from internal variability, and should apply this framework to other vulnerable coastal regions. Integrating such projections with urban-scale simulations, which can capture city-level amplification through the urban heat island effect (*36*), will also be important for translating climate signals into estimates of human exposure and adaptation needs.

In conclusion, our results show that kilometer-scale resolution can substantially change how coastal humid-heat extremes are represented. The key implication is not that all kilometer-scale models are automatically more reliable, but that coarse-resolution climate information can smooth out localized extremes in precisely the places where human exposure is highest, thus understating the potential risk that future humid-heat extreme changes pose to societies located along humid, hot coastal zones. In particular, our results suggest that current projections may underestimate the number of people who will be exposed to levels of humid heat beyond what is considered safe, thus placing greater urgency on the need for climate adaptation in vulnerable regions.

## MATERIALS AND METHODS

### Research area

The whole selected domain extends from 10°N to 35°N latitude and 30°E to 65°E longitude (Fig. 9), covering a diverse range of geographical features such as coasts, deserts, and steep mountain ranges. Within this area, two polygonal masks (drawn in blue and red in Fig. 9) were designed to focus the Extreme Days detection on the two seas and their nearby coasts.

**Fig. 9.**
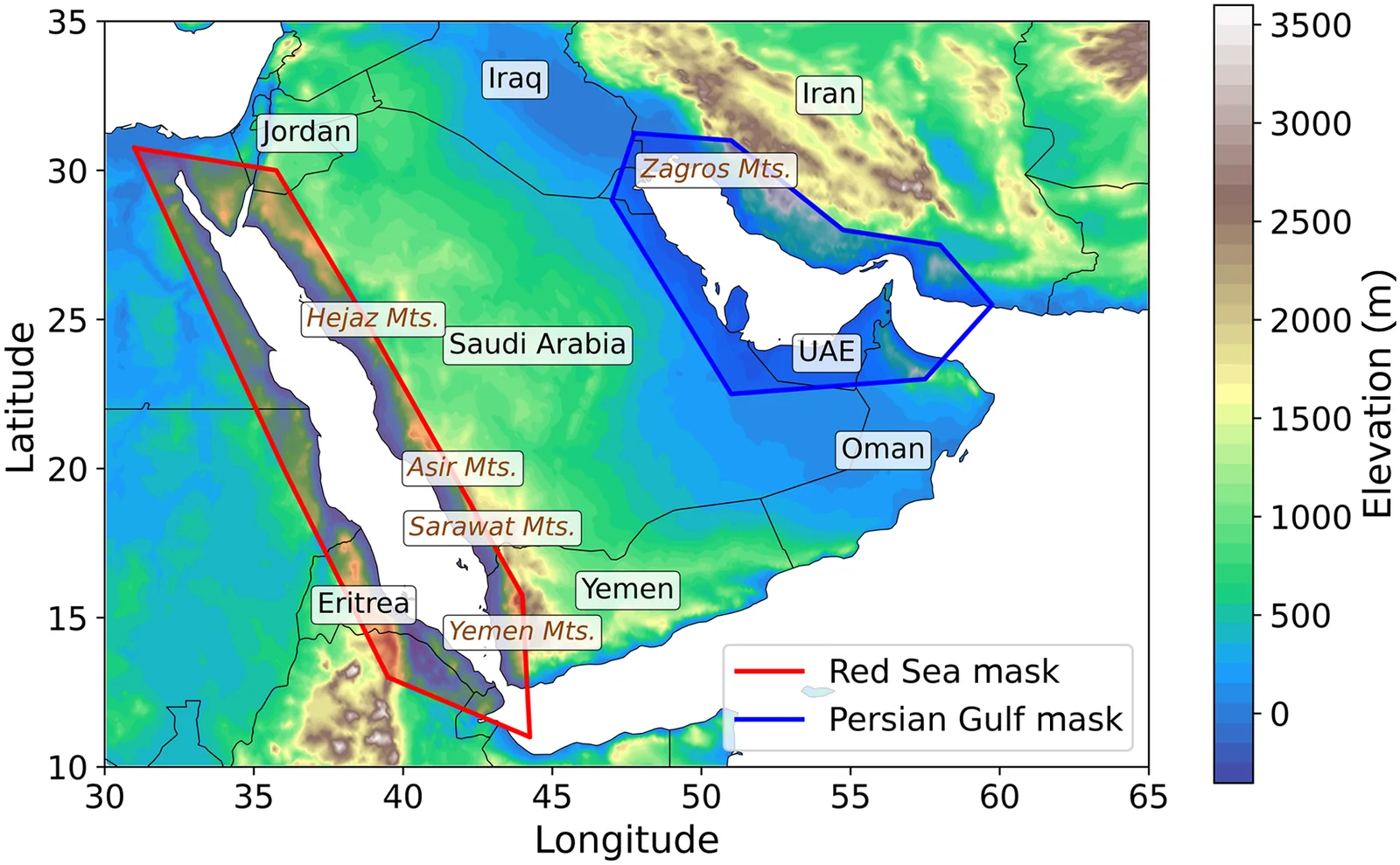
Research area, its topographic elevation in meters, and the two polygonal masks of the Red Sea and Persian Gulf. Coastlines and national boundaries are from Natural Earth (public domain).

### Data

The study uses four primary sources of data: nextGEMS model outputs (*19*), ERA5 reanalysis (*20*), and the Global Historical Climatology Network hourly [GHCNh; (*21*)] data as observational basis and CMIP6 models as benchmark.

Within the nextGEMS project, two fully coupled atmosphere-ocean ESMs were developed. These incorporate the ICON Weather and Climate Model in its Sapphire configuration, developed by the Max Planck Institute for Meteorology (*37*), and the IFS coupled to the FESOM (*38*, *39*), developed by ECMWF (*40*, *41*). In this work, we used the latest nextGEMS production runs, spanning 30 years (2020 to 2049), with both models’ transient runs following the SSP3-7.0 scenario (*42*). IFS-FESOM also provides a historical simulation covering 1990 to 2019, which will be used for validation purposes.

While the full period extends from 2020 to 2049, we discard the first 4 years of the ICON simulation due to a spurious negative temperature drift during this initial spin-up phase. As a result, all analyses focus on the period 2024 to 2049.

The two models have the following characteristics: IFS runs on a 9-km-resolution grid, coupled with the FESOM ocean module, operating at an eddy-resolving resolution of 5 km and providing essential surface boundary conditions, including SST, sea ice concentration, and zonal/meridional surface currents. The land component is represented by ECLand (*43*), while the ecWAM wave model (*44*) accounts for ocean surface wave interactions. IFS solves hydrostatic equations and includes advanced parameterizations for convection, radiation, turbulence, cloud microphysics, and orographic drag.

In contrast, ICON uses a 10-km icosahedral-triangular C-grid (*45*) for the atmospheric component and is formulated using nonhydrostatic equations. Unlike IFS, ICON does not use a parameterization scheme for convection.

Its ocean component (ICON-O) operates at a 5-km resolution, ensuring high-resolution coupling with the atmosphere. The land component is represented by the Jena Scheme for Biosphere-Atmosphere Coupling in Hamburg (*46*), which facilitates interactions with the ocean via hydrological discharge.

In both simulations, the outputs are written on HEALPix grids (*24*), consisting of a quadrilateral-based pixelation of a spherical shape. This grid system divides the Earth’s surface into 12 × 4*^z^* equal-area cells, where *z* is the resolution parameter, called zoom level hereon. The resolution corresponding to each zoom level involved in this study is summarized in Table 1 (*23*).

**Table 1.** HEALPix grid resolutions at different zoom levels.

HEALPix z	Angular resolution (°)	Linear resolution (km)	Grid cells (*N*_pix_)
**zoom5**	∼1.4	∼204	12,288
**zoom7**	∼0.35	∼51	196,608
**zoom9**	∼0.09	∼13	3,145,728

For the historical validation, we used the period 1990 to 2019, comparing TW from ERA5 and the IFS-FESOM historical run against GHCNh station data. Because a historical simulation is not available for ICON, this validation is limited to IFS-FESOM and ERA5.

From the GHCNh stations available in the study region, we selected those with at least 10 years of data, with at least 360 valid days per year, after 1990 and for each of the remaining ones, the closest grid point in both ERA5 and IFS-FESOM is selected without interpolation, ensuring a consistent spatial comparison. Out of the 597 stations available in the study region, 85 were retained based on this criterion.

For the computation of TW, in all the three data sources, (see the TW computation section) the variables involved are the surface pressure (*p*), specific humidity at 2 m (*q*_v_), 2-m temperature (*T*), and 2-m dew point temperature (*T*_d_). To inspect the typical conditions related to the highest TW values, the two components of wind speed (*u*_10_, *v*_10_) are extracted as well. The datasets, their selected output variables, and their temporal resolution available are summarized in Table 2.

**Table 2.** Overview of the model variables and their temporal resolutions.

Source	Variables	Time step
**ICON**	*p*, *q*_v_, *T*, *u*_10_, *v*_10_	3-Hourly
**IFS-FESOM**	*p*, *T*, *T*_d_, *u*_10_, *v*_10_	Hourly
**ERA5**	*p*, *T*, *T*_d_, *u*_10_, *v*_10_	Hourly
**GHCNh**	*p*, *T*, *T*_d_	Hourly
**CMIP6**	TW metric	Daily

### TW computation

Several methods have been proposed for the calculation of TW [see (*47*)]; the choice is strongly dependent on data availability, and greatly varies across studies (*48*, *49*). The one from (*50*), considered the most accurate, was chosen to carry out this study, adopting the formulation introduced by Buzan *et al.* (*47*).

The computation proceeds as follows: First, saturation vapor pressure is estimated, based on the Clausius-Clapeyron relationship and actual vapor pressure is then computed from either *T*_d_ or *q*_v_, depending on variable availability (see Table 2). Relative humidity (RH) is then calculated as

$$\text{RH}=\frac{e}{e_{s}}\times 100$$ (1)

where *e*/*e*_s_ is the ratio of the water vapor pressure and the saturation water vapor pressure. When *T*_d_ is not available (e.g., ICON), actual vapor pressure is obtained via

$$e=\frac{q_{v}\cdot p}{\varepsilon +q_{v}\cdot \left(1-\varepsilon \right)}$$ (2)

with ε assumed equal to 0.622.

The Davies-Jones iterative routine is then grounded in the theory of equivalent potential temperature (θ_e_). The method simulates the adiabatic ascent of an air parcel to a high altitude, during which all water vapor condenses and releases latent heat, and the resulting condensate is removed via precipitation. The parcel is then brought dry-adiabatically back down to a reference pressure of 1000 hPa and this yields the wet-bulb potential temperature (θ_w_), which is equivalent to θ_e_.

TW at the original pressure level *p* is then obtained by following an isoline of θ_w_ downward, identifying the temperature at which the air parcel would have that same θ_w_ at the current pressure. This procedure can be expressed implicitly as solving for TW such that

$$\theta_{w}\left(\text{TW},p\right)=\theta_{e}$$

where θ_w_ is computed via an iterative moist thermodynamic routine accounting for latent heat release, saturation processes, and adiabatic transformations.

The method was applied consistently across all datasets at their highest temporal resolution. Most of the results presented show comparisons using the daily maximum TW obtained after its computation: for IFS-FESOM, GHCNh, and ERA5, *T*, *T*_d_, and *p* were used directly, In the case of ICON, where *q*_v_ is provided instead of *T*_d_, we computed *e* and RH as intermediate steps before passing the data to the TW solver.

### CMIP6 benchmark

To place the kilometer-scale simulations in the context of conventional coarse-resolution climate models, we compared ICON and IFS-FESOM with raw CMIP6 simulations over the AP domain. This comparison is intended as an independent benchmark, rather than as a controlled resolution experiment, because CMIP6 models differ from nextGEMS not only in grid spacing, but also in model formulation, parameterizations, and mean-state biases.

We used one realization from each CMIP6 model providing TW under the SSP3-7.0 scenario. To ensure consistency with the available CMIP6 heat-stress product, TW from ICON and IFS-FESOM was recomputed following the same daily input convention used in the CMIP6 product: daily maximum near-surface temperature, daily mean near-surface specific humidity, and daily mean surface pressure. For IFS-FESOM, near-surface–specific humidity was derived from dew-point temperature and surface pressure, while ICON-specific humidity was taken directly from the model output. TW was then calculated using the same Davies-Jones formulation described above.

CMIP6 models were used in raw form, rather than bias-adjusted form, to avoid comparing bias-adjusted CMIP6 fields with unadjusted nextGEMS simulations. Unless stated otherwise, all nextGEMS, ERA5, and GHCNh TW diagnostics are computed at the native temporal resolution of each dataset and then converted to daily maximum TW. The only exception is the comparison with CMIP6, where ICON and IFS-FESOM TW are recomputed following the daily input convention of the available CMIP6 heat-stress product.

### Methods of aggregation to coarser resolution

A major advantage of using HEALPix-based outputs is the ability to seamlessly adjust resolution levels, and this flexibility allows for reducing the resolution by aggregating grid cells (*24*). For example, transitioning from two consecutive zoom levels involves averaging the values of four adjacent grid cells from the higher resolution to create a single representative value at the lower resolution. This approach will be referred to as the “MEAN method” (Fig. 10).

**Fig. 10.**
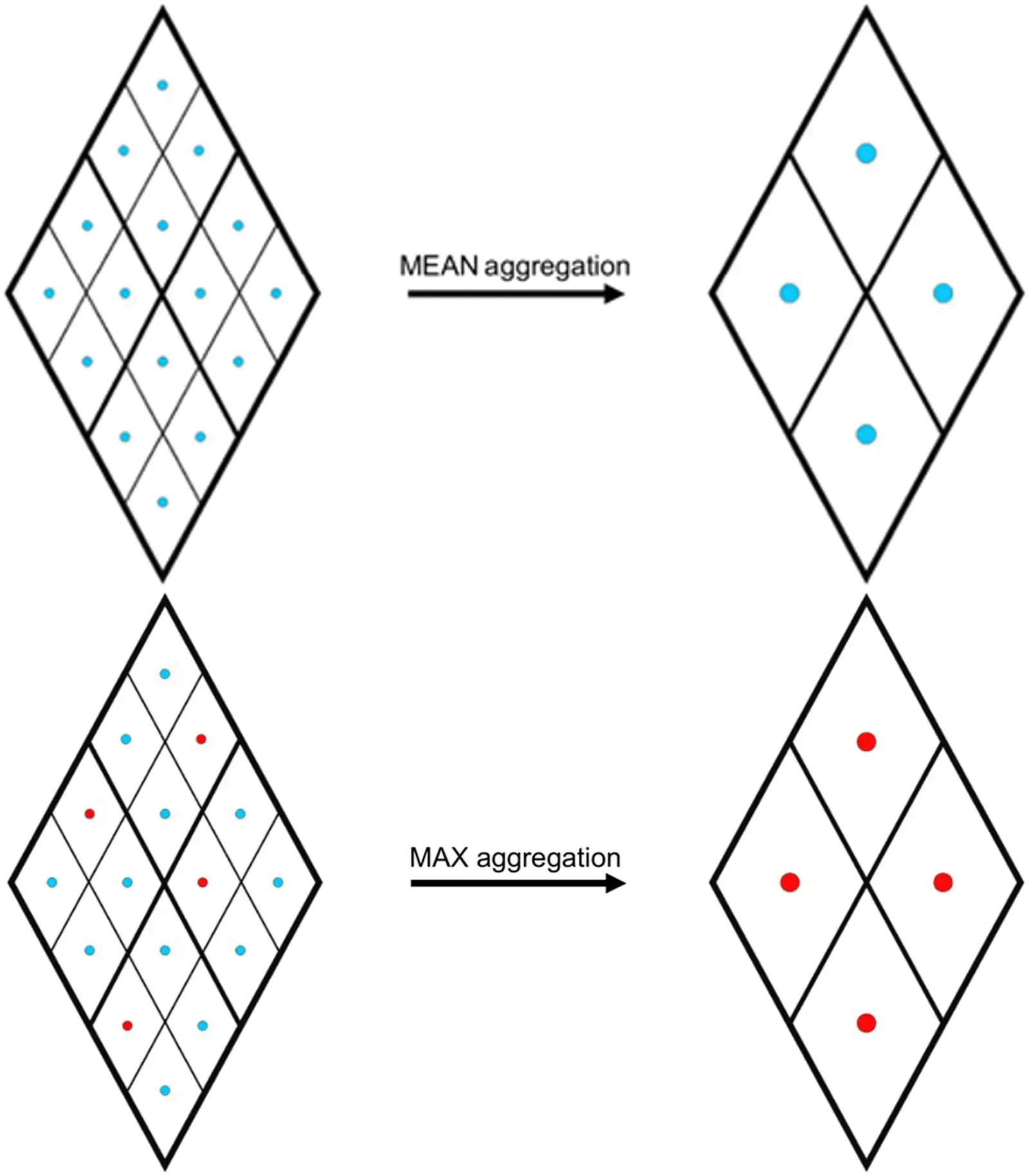
Aggregation with MEAN and MAX methods.

To evaluate whether higher-resolution simulations capture localized maxima with greater intensity, we introduce an aggregation technique called the “MAX method.” The MAX method identifies the maximum value among the four finer-resolution cells (red dots in Fig. 10) corresponding to a single coarser-resolution cell. The highest value is then assigned as the representative of that grid cell in the lower-resolution dataset. By doing so, the method effectively retains the most extreme values detected within the high-resolution field while ensuring a fair comparison with coarser grids.

These two techniques were applied in two declinations:

1) The single variables from the models’ outputs required to calculate TW are aggregated from zoom9 (13 km) to zoom7 (51 km) and further to zoom5 (204 km) using the MEAN method. TW is then recomputed from the aggregated variables in the three different resolutions.

2) The TW fields computed in the zoom9 resolution (i.e., the highest resolution) are aggregated directly first to zoom7 and second to zoom5 with the MAX method. This strategy retains the maxima simulated in the highest resolution and compares them to the ones obtained in point 1.

## Supplementary Materials

### This PDF file includes:

Supplementary Text Figs. S1 to S5 References
