# Supplementary material for: Coarse climate model grids underestimate coastal humid-heat extremes
Source: Sci Adv. 2026 Oct 30;12(44):eael4020. doi: 10.1126/sciadv.ael4020 (PMC13721960; doi:10.1126/sciadv.ael4020)
Supplement: Supplementary file 1 [file sciadv.ael4020_sm.pdf]

Science Advances
AAAS

Supplementary Materials for

Coarse climate model grids underestimate coastal humid-heat extremes

Cosimo E. Carniel *et al.*

Corresponding author: Cosimo E. Carniel, cosimo.carniel@env.ethz.ch

Sci. Adv. **12**, eael4020 (2026)
DOI: 10.1126/sciadv.ael4020

This PDF file includes:

Supplementary Text
Figs. S1 to S5
References

Extreme Days detection

Once TW is calculated in every dataset at the highest temporal resolutions, a systematic detection of "Extreme Days" (EDs) is applied following this logic:

1. The 99th percentile fields of TW (in the highest temporal resolution of each model considered) are computed for the entire 2024-2049 period.
2. The daily maximum TW values are selected.
3. For every grid cell and day, we determine if the daily maximum TW exceeds the local 99th percentile of TW. This step is performed using CDO (Climate Data Operators), and the result is a mask holding 1 if the grid cell satisfies the condition, and 0 otherwise.
4. Two polygonal masks are created "ad hoc" for the areas around the two main bodies of water (Fig. 9) and an ED is detected if, on such a day, at least 50% of the grid cells inside the masks exceed the local 99th percentile of TW.

This process produces, for each dataset, two sets of EDs, one for each separate mask; hence, the individual days might differ. Nevertheless, since the study is focused on how the models represent the coastal contributions to humid heat stress, and not strictly on the specific days' identification, we choose to employ this double mask strategy. Furthermore, if we considered a threshold of 50% in the entire region, we would have to take into account also the vast desert area between the two seas, where, despite the high temperatures, the humidity is very low and would not result in high TW values, thus leading to a possible underestimation of the number of EDs even along the coasts.

As a final step, the fields of T, u_{10}, v_{10} and RH are extracted within the region of interest during every timestep available in the EDs and averaged over the time dimension, to conduct a composite analysis of each variable and understand the patterns leading to the extreme TW values recorded in these instances.

Composite analysis

As detailed above, the identification of EDs is performed separately for the Red Sea and Persian Gulf regions, hence, for each dataset analyzed (ICON, IFS-FESOM, and ERA5), two distinct lists of EDs are generated. The objective of the composite analysis is to determine what average regional patterns appear during these days, but we are also interested in understanding whether these patterns differ when contributing to high TW occurrences in one region rather than the other. After this experiment was conducted, we concluded that the patterns leading to the two sets of days are in fact very similar. Therefore, to focus on the comparison across the models rather than a geographical one, some of the following plots illustrate the results of the composite analysis for Red Sea's EDs

Figure S1 summarizes the thermal, moisture, and wind patterns accompanying extreme humid heat events in the Red Sea. Temperatures consistently exceed 30°C, reaching above 34°C in places, while relative humidity remains high (70-95%), underscoring the joint role of heat and moisture in driving TW extremes. Both km-scale models and ERA5 capture strong coastal moisture advection, with RH above 80% over water and adjacent land, extending farther inland in ICON. Wind fields

show weak onshore flow (2-4 m/s) converging with desert air masses along the Red Sea and Persian Gulf coasts, conditions that trap humidity and reduce atmospheric mixing.

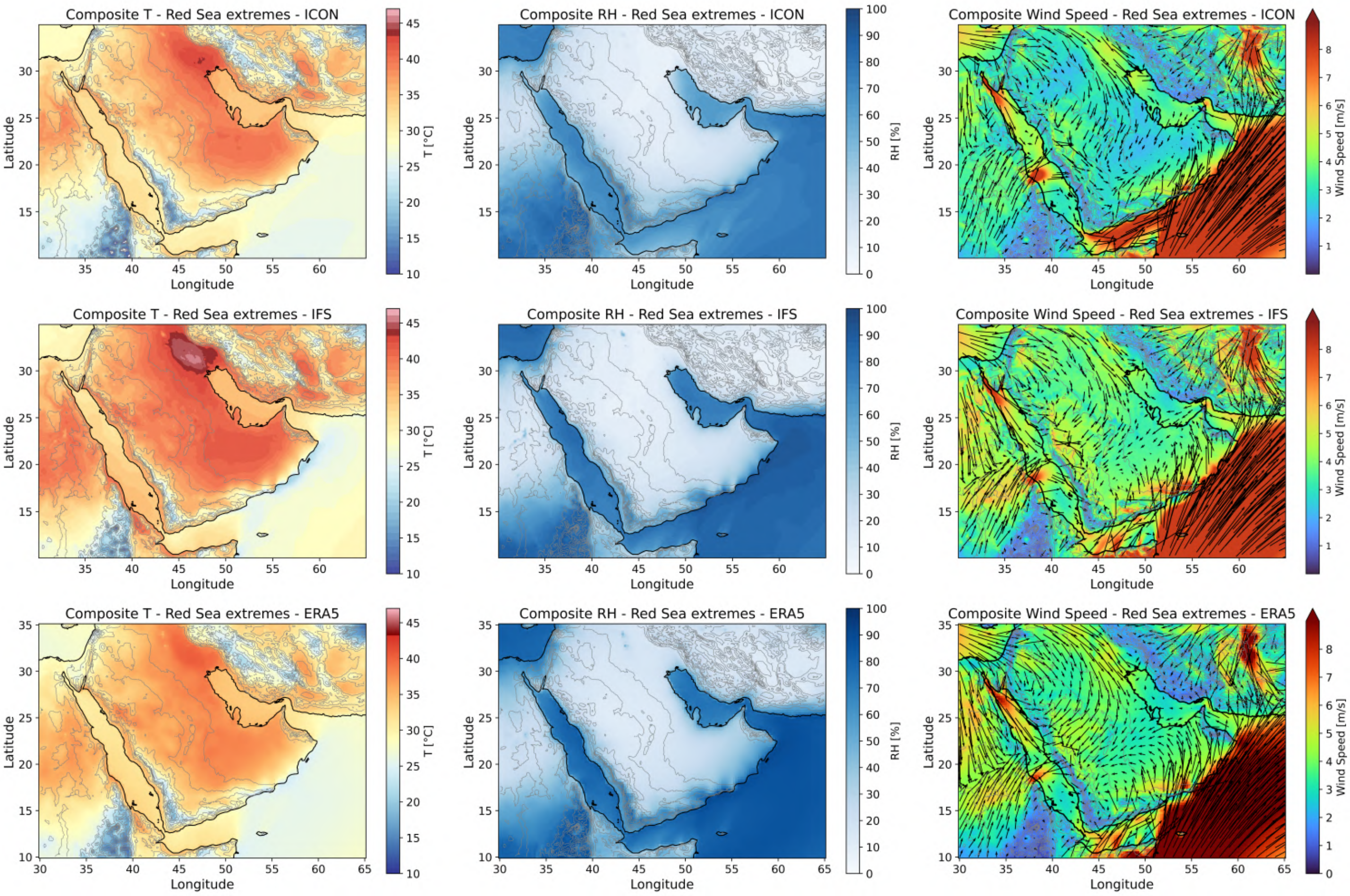

Figure S1: Composite meteorological fields during EDs detected over the Red Sea mask. The panels show temperature (T, left column, in °C), relative humidity (RH, center column, in %), and wind speed (right column, in m/s, with vectors representing wind direction). Rows correspond to different datasets: ICON (top), IFS-FESOM (middle), and ERA5 (bottom).

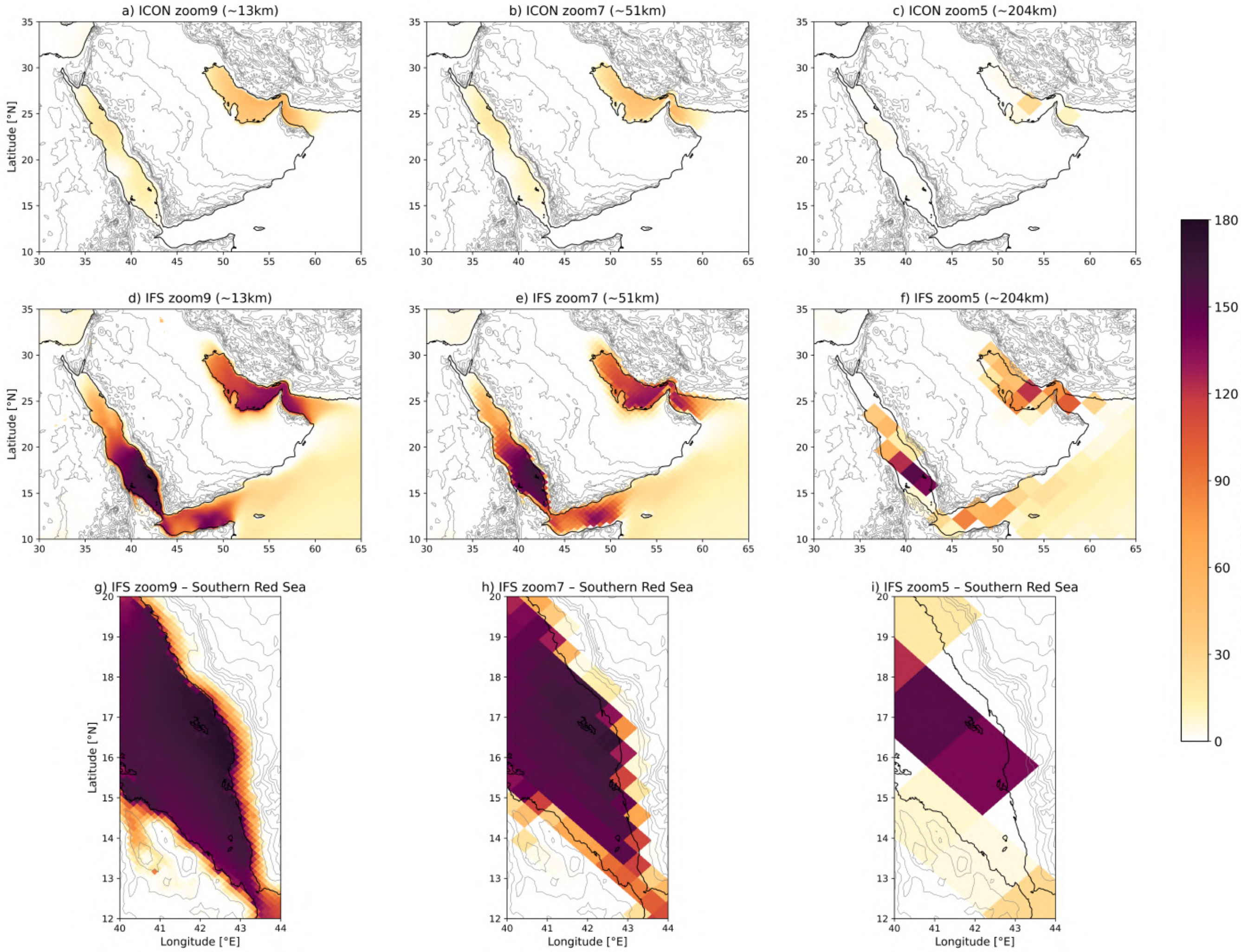

Figure S2: Frequency and persistence of humid heat extremes strongly underestimated at coarser resolution. Average annual exceedances of the 28 °C TW threshold during 2024-2049. Panels show the average number of days per year exceeding 28 °C in ICON **(a-c)** and IFS-FESOM **(d-f, bottom row)** at zoom9 (left), zoom7 (middle), and zoom5 (right) resolutions. Exceedances in the coarser grids (zoom7 and zoom5) are derived by aggregating the input variables for the computation of TW using the MEAN method; **g, h** and **i** provide a zoomed-in view of the southern Red Sea coast of d, e and f

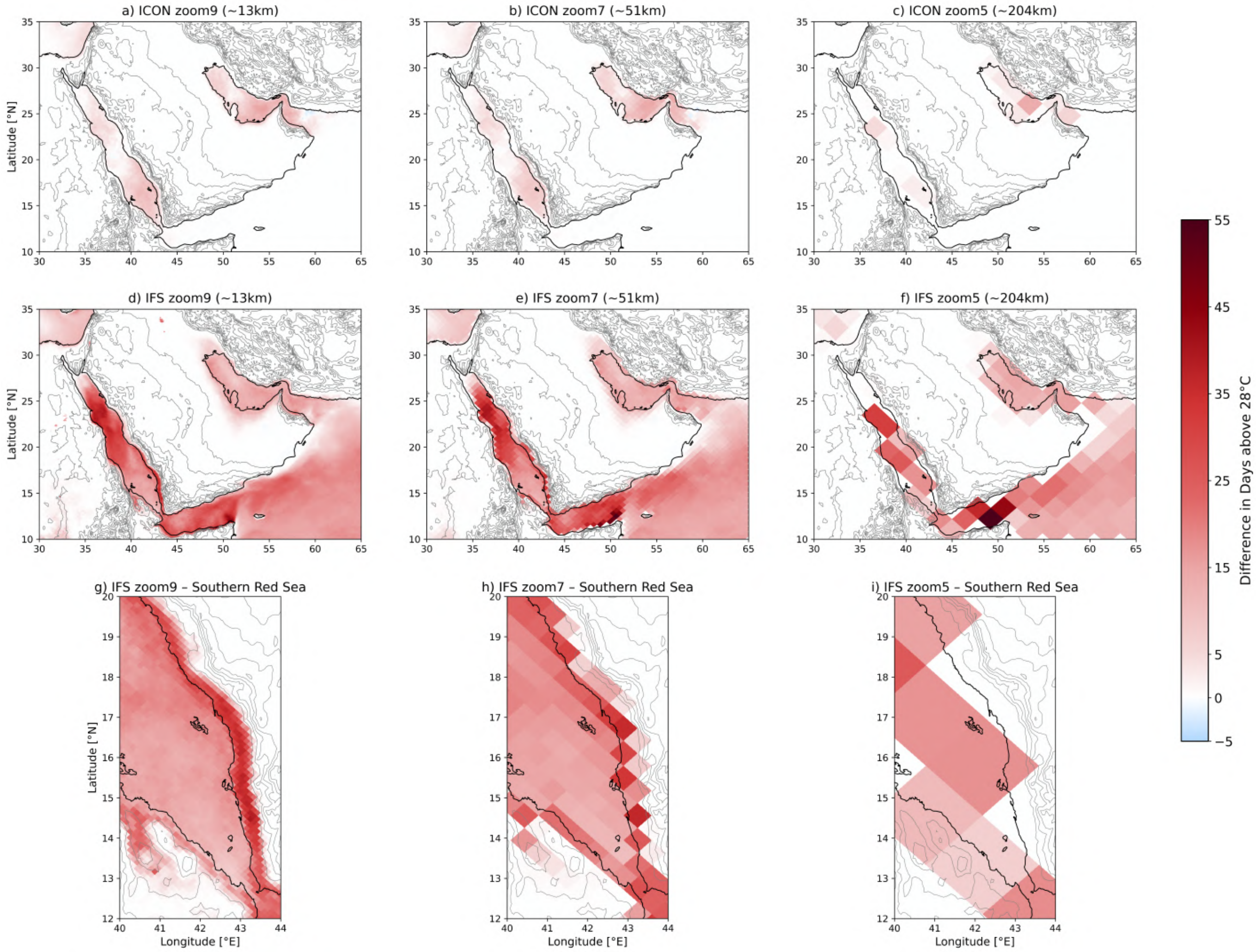

Figure S3: Stronger change in humid-heat exceedance frequency at km-scale resolution. Difference in the average number of days per year with TW>28°C between the periods 2037-2049 and 2024-2036 across different resolutions of ICON **(a-c)** and IFS-FESOM **(d-f, bottom row)** at zoom9 (left), zoom7 (middle), and zoom5 (right) resolutions. Exceedances in the coarser grids (zoom7 and zoom5) are derived by aggregating the input variables for the computation of TW using the MEAN method. **g, h** and **i** provide a zoomed-in view of the southern Red Sea coast of d, e and f

Trend estimation and significance

To investigate the spatially distributed trends of TW during the summer (JJA) season, we apply the trend-free pre-whitened Mann-Kendall test, proposed by (51), a non-parametric method widely used to detect monotonic trends while correcting for potential autocorrelation (3). The magnitude of the trend is then quantified using Sen's slope estimator at each grid cell, and the statistical significance is assessed through the test's associated p-values. Trends are considered significant at the 5% level ($p \leq 0.05$).

This method is also used to separate the contributions of T and q_v to the variability and trend of the TW. This decomposition is performed by comparing the computed trends of TW in three different configurations:

1. Natural trend of TW: The standard trend computed using the full JJA dataset, where both T and q_v vary as simulated in the model run. This reflects the combined influence of temperature and humidity on TW over time.

2. Trend of TW with constant q_v: TW is recalculated at each time step by keeping specific humidity q_v fixed at its long-term summer (JJA) mean over the period 2024-2049, while allowing T to vary. This isolates the influence of temperature changes on TW, assuming no change in humidity.

3. Trend of TW with constant T: TW is recalculated by keeping T fixed at its long-term summer mean, while allowing q_v to vary. This isolates the contribution of humidity variability to changes in TW.

It is important to note that, in point 3., T is fixed both in the calculation of RH (Eq. 1) and in the one of TW. By doing so, we intentionally remove the thermodynamic dependence between T and q_v that is governed by the Clausius-Clapeyron (C-C) law.

In the real atmosphere, warming increases the saturation vapor pressure exponentially, and, under the assumption of approximately constant RH (52), this leads to a corresponding increase in q_v. However, by fixing T, the saturation specific humidity remains constant, and q_v is no longer influenced by changes in T. Consequently, T and q_v are treated as independent variables in this setup.

This decoupling breaks the typical C-C scaling mechanism and allows us to isolate the influence of atmospheric moisture changes alone on TW. In particular, because RH in our framework is diagnosed from time-varying q_v and fixed T, any changes in RH, and thus in TW, are driven solely by variations in q_v that are not directly linked to temperature increases.

The trend-decomposition diagnostics indicate that the simulated increase in summer daily maximum TW is mainly moisture-driven in both km-scale models. In ICON, increasing specific humidity explains about 60% of the area-mean TW trend, while temperature accounts for roughly 30%. IFS-FESOM shows a similar qualitative behaviour, with humidity also contributing around 60% of the total trend.

Spatially, one notable feature in ICON is the muted TW trend in the Tokar Gap Jet region (53), likely because enhanced ventilation limits moisture accumulation over the adjacent Red Sea. Overall, these supplementary diagnostics support the interpretation that future intensification of Arabian Peninsula humid heat is strongly controlled by increasing near-surface moisture.

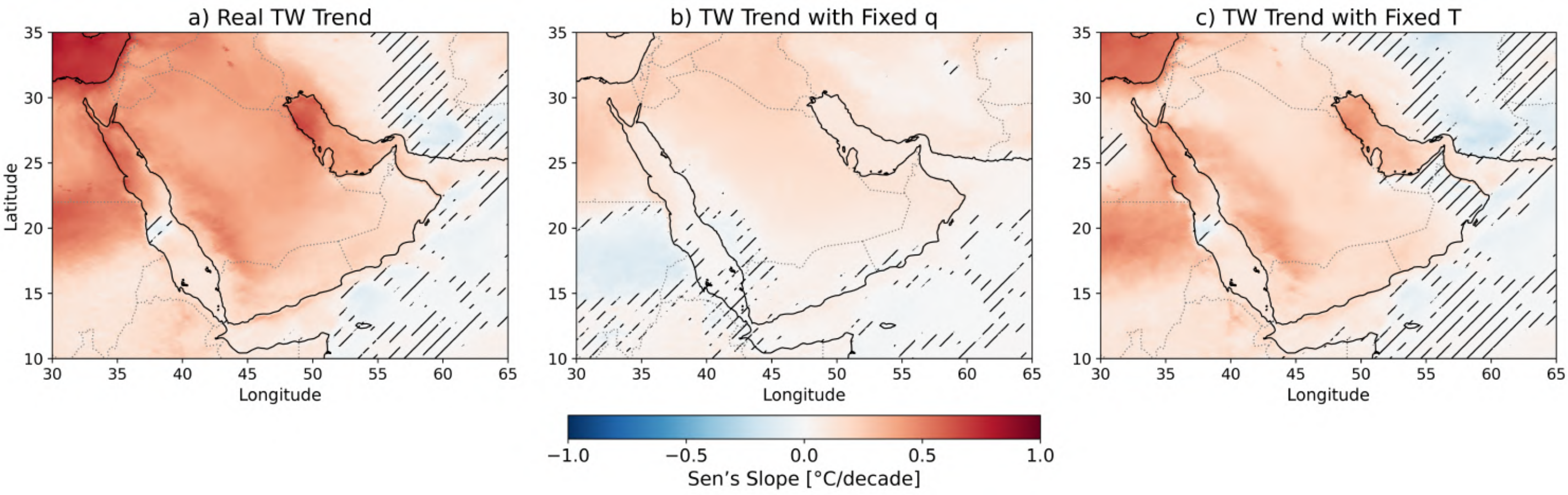

Figure S4: Contributions of temperature and humidity to summer daily maximum TW trends over the AP based on ICON simulations. **a)** Actual TW trend computed from the full simulations; **b)** TW trend computed by fixing q_v, isolating the contribution of temperature; **c)** TW trend computed by fixing T, isolating the contribution of humidity. Sen's slope was computed on the daily summer (JJA) TW time series. Since the analysis includes only summer days, the resulting slope (°C per day) was multiplied by 920 days to express the trend in °C/decade. The colorbar indicates the magnitude of the trend, while hatched areas denote regions where the trend is not statistically significant (at the 5% level).

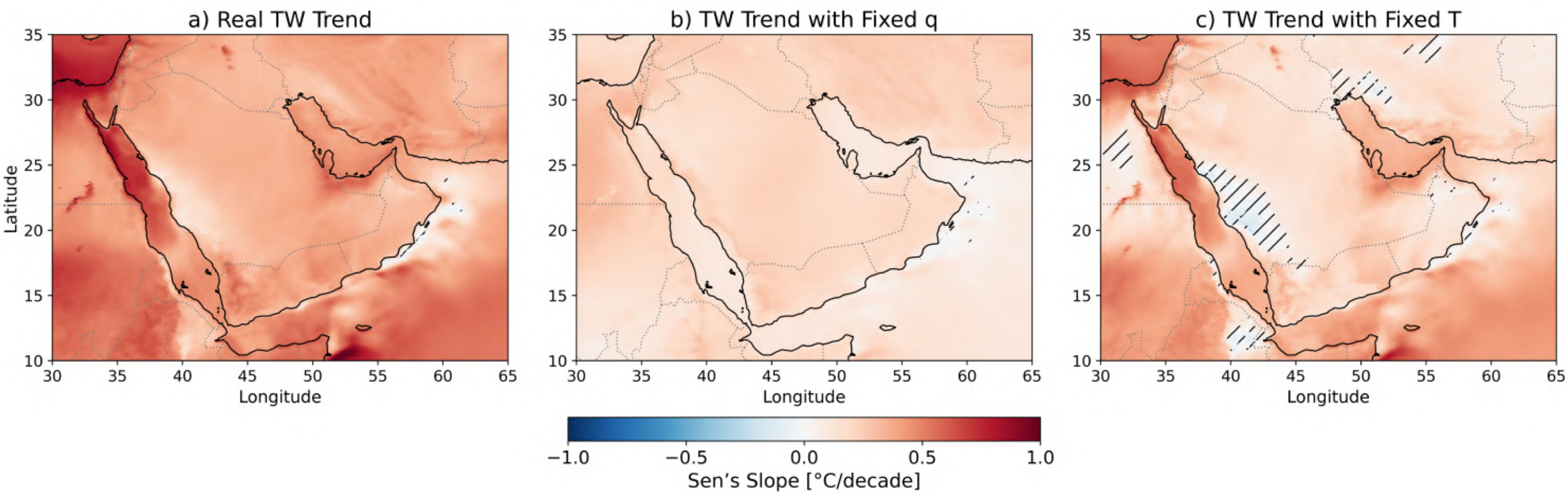

Figure S5: Contributions of temperature and humidity to summer daily maximum TW trends over the AP based on IFS-FESOM simulations. **a)** Actual TW trend computed from the full simulations; **b)** TW trend computed by fixing q_v, isolating the contribution of temperature; **c)** TW trend computed by fixing T, isolating the contribution of humidity. Sen's slope was computed on the daily summer (JJA) TW time series. Since the analysis includes only summer days, the resulting slope (°C per day) was multiplied by 920 days to express the trend in °C/decade. The colorbar indicates the magnitude of the trend, while hatched areas denote regions where the trend is not statistically significant (at the 5% level).

REFERENCES

1. D. García-León, A. Casanueva, G. Standardi, A. Burgstall, A.D. Flouris, L. Nybo, Current and projected regional economic impacts of heatwaves in Europe. *Nat. Commun.* **12**, 5807 (2021).

2. S. E. Perkins-Kirkpatrick, C. H. Gregory, J. K. Vanos, J. W. Baldwin, H. Staudmyer, G. Guzman-Echavarria, O. Jay, Deadly heat stress conditions are already occurring. *Nat. Commun.* **17**, 2590 (2026).

3. Q. Ma, Y. Chen, M. Ionita, European summer wet-bulb temperature: Spatiotemporal variations and potential drivers. *J. Climate* **37**, 2059–2080 (2024).

4. G. Zittis, M. Almazroui, P. Alpert, P. Ciais, W. Cramer, Y. Dahdal, M. Fnais, D. Francis, P. Hadjinicolaou, F. Howari, A. Jrrar, D. G. Kaskaoutis, M. Kulmala, G. Lazoglou, N. Mihalopoulos, X. Lin, Y. Rudich, J. Sciare, G. Stenchikov, E. Xoplaki, J. Lelieveld, Climate change and weather extremes in the Eastern Mediterranean and Middle East. *Rev. Geophys.* **60**, e2021RG000762 (2022).

5. N. S. Diffenbaugh, F. Giorgi, Climate change hotspots in the CMIP5 global climate model ensemble. *Clim. Change* **114**, 813–822 (2012).

6. P. Patlakas, C. Stathopoulos, H. Flocas, C. Kalogeri, G. Kallos, Regional climatic features of the Arabian Peninsula. *Atmosphere* **10**, 220 (2019).

7. J. Pal, E. Eltahir, Future temperature in southwest Asia projected to exceed a threshold for human adaptability. *Nat. Clim. Change* **6**, 197–200 (2016).

8. C. Raymond, T. Matthews, R. M. Horton, E. M. Fischer, S. Fueglistaler, C. Ivanovich, L. Suarez-Gutierrez, Y. Zhang, On the controlling factors for globally extreme humid heat. *Geophys. Res. Lett.* **48**, e2021GL096082 (2021).

9. Y. Alosairi, N. Alsulaiman, A. Rashed, D. Al-Houti, World record extreme sea surface temperatures in the northwestern Arabian/Persian Gulf verified by in situ measurements. *Mar. Pollut. Bull.* **161**, 111766 (2020).

10. C. Raymond, T. Matthews, R. M. Horton, The emergence of heat and humidity too severe for human tolerance. *Sci. Adv.* **6**, eaaw1838 (2020).

11. J. Vanos, G. Guzman-Echavarria, J. W. Baldwin, C. Bongers, K. L. Ebi, O. Jay, A physiological approach for assessing human survivability and liveability to heat in a changing climate. *Nat. Commun.* **14**, 7653 (2023).

12. S. Speizer, C. Raymond, C. Ivanovich, R. M. Horton, Concentrated and intensifying humid heat extremes in the IPCC AR6 regions. *Geophys. Res. Lett.* **49**, e2021GL097261 (2022).

13. J. Lelieveld, Y. Proestos, P. Hadjinicolaou, M. Tanarhte, E. Tyrlis, G. Zittis, Strongly increasing heat extremes in the Middle East and North Africa (MENA) in the 21st century. *Clim. Change* **137**, 245–260 (2016).

14. M. Almazroui, Changes in temperature trends and extremes over Saudi Arabia for the period 1978–2019. *Adv. Meteorol.* **2020**, 8828421 (2020).

15. A. M. El Kenawy, J. I. Lopez-Moreno, M. F. Mc Cabe, S. M. Robaa, F. Domínguez-Castro, M. Peña-Gallardo, R. M. Trigo, M. E. Hereher, T. Al-Awadhi, S. M. Vicente-Serrano, Daily temperature extremes over Egypt: Spatial patterns, temporal trends, and driving forces. *Atmos. Res.* **226**, 219–239 (2019).

16. B. H. Samset, C. Zhou, J. S. Fuglestvedt, M. T. Lund, J. Marotzke, M. D. Zelinka, Steady global surface warming from 1973 to 2022 but increased warming rate after 1990. *Commun. Earth Environ.* **4**, 400 (2023).

17. F. Li, T. Tamarin-Brodsky, Atmospheric stability sets maximum moist heat and convection in the midlatitudes. *Sci. Adv.* **12**, eaea8453 (2026).

18. A. Arakawa, The cumulus parameterization problem: Past, present, and future. *J. Climate* **17**, 2493–2525 (2004).

19. H. Segura, X. Pedruzo-Bagazgoitia, P. Weiss, S. K. Müller, T. Rackow, J. Lee, E. Dolores-Tesillos, I. Benedict, M. Aengenheyster, R. Aguridan, G. Arduini, A. J. Baker, J. Bao, S. Bastin,

E. Baulenas, T. Becker, S. Beyer, H. Bockelmann, N. Brüggemann, L. Brunner, S. K. Cheedela, S. Das, J. Denissen, I. Dragaud, P. Dziekan, M. Ekblom, J. F. Engels, M. Esch, R. Forbes, C. Frauen, L. Freischem, D. García-Maroto, P. Geier, P. Gierz, Á. González-Cervera, K. Grayson, M. Griffith, O. Gutjahr, H. Haak, I. Hadade, K. Haslehner, S. u. Hasson, J. Hegewald, L. Kluft, A. Koldunov, N. Koldunov, T. Kölling, S. Koseki, S. Kosukhin, J. Kousal, P. Kuma, A. U. Kumar, R. Li, N. Maury, M. Meindl, S. Milinski, K. Mogensen, B. Niraula, J. Nowak, D. S. Praturi, U. Proske, D. Putrasahan, R. Redler, D. Santuy, D. Sármány, R. Schnur, P. Scholz, D. Sidorenko, D. Spät, B. Sützl, D. Takasuka, A. Tompkins, A. Uribe, M. Valentini, M. Veerman, A. Voigt, S. Warnau, F. Wachsmann, M. Wacławczyk, N. Wedi, K.-H. Wieners, J. Wille, M. Winkler, Y. Wu, F. Ziemen, J. Zimmermann, F. A.-M. Bender, D. Bojovic, S. Bony, S. Bordoni, P. Brehmer, M. Dengler, E. Dutra, S. Faye, E. Fischer, C. van Heerwaarden, C. Hohenegger, H. Järvinen, M. Jochum, T. Jung, J. H. Jungclaus, N. S. Keenlyside, D. Klocke, H. Konow, M. Klose, S. Malinowski, O. Martius, T. Mauritsen, J. P. Mellado, T. Mieslinger, E. Mohino, H. Pawłowska, Karsten Peters-von Gehlen, A. Sarré, P. Sobhani, P. Stier, L. Tuppi, P. L. Vidale, I. Sandu, B. Stevens, nextGEMS: Entering the era of kilometer-scale Earth system modeling. *EGUsphere* 1–39 (2025).

20. H. Hersbach, B. Bell, P. Berrisford, S. Hirahara, A. Horányi, J. Muñoz-Sabater, J. Nicolas, C. Peubey, R. Radu, D. Schepers, A. Simmons, C. Soci, S. Abdalla, X. Abellan, G. Balsamo, P. Bechtold, G. Biavati, J. Bidlot, M. Bonavita, G. de Chiara, P. Dahlgren, D. Dee, M. Diamantakis, R. Dragani, J. Flemming, R. Forbes, M. Fuentes, A. Geer, L. Haimberger, S. Healy, R. J. Hogan, E. Hólm, M. Janisková, S. Keeley, P. Laloyaux, P. Lopez, C. Lupu, G. Radnoti, P. de Rosnay, I. Rozum, F. Vamborg, S. Villaume, J.N. Thépaut, The ERA5 global reanalysis. *Q. J. Roy. Meteorol. Soc.* **146**, 1999–2049 (2020).

21. R. J. H. Dunn, K. M. Willett, D. E. Parker, L. Mitchell, Expanding HadISD: Quality-controlled, sub-daily station data from 1931. *Geosci. Instrum. Methods Data Syst.* **5**, 473–491 (2016).

22. N. Freychet, S. Tett, Z. Yan, Z. Li, Underestimated change of wet-bulb temperatures over east and south China. *Geophys. Res. Lett.* **47**, e2019GL086140 (2020).

23. L. Brunner, B. Poschlod, E. Dutra, E. M. Fischer, O. Martius, J. Sillmann, A global perspective on the spatial representation of climate extremes from km-scale models. *Environ. Res. Lett.* **20**, 074054 (2025).

24. K. M. Górski, E. Hivon, A. J. Banday, B. D. Wandelt, F. K. Hansen, M. Reinecke, M. Bartelmann, HEALPix: A framework for high-resolution discretization and fast analysis of data distributed on the sphere. *Astrophys. J.* **622**, 759–771 (2005).

25. D. J. Vecellio, Q. Kong, W. L. Kenney, M. Huber, Greatly enhanced risk to humans as a consequence of empirically determined lower moist heat stress tolerance. *Proc. Natl. Acad. Sci. U.S.A.* **120**, e2305427120 (2023).

26. E. M. Fischer, K. W. Oleson, D. M. Lawrence, Contrasting urban and rural heat stress responses to climate change. *Geophys. Res. Lett.* **39**, L03705 (2012).

27. S. Ullah, A. Aldossary, W. Ullah, S. G. Al-Ghamdi, Augmented human thermal discomfort in urban centers of the Arabian Peninsula. *Sci. Rep.* **14**, 3974 (2024).

28. N. Odnoletkova, T. W. Patzek, Data-driven analysis of climate change in Saudi Arabia: Trends in temperature extremes and human comfort indicators. *J. Appl. Meteorol. Climatol.* **60**, 1055–1070 (2021).

29. J. Liu, H. Kim, M. Hashizume, W. Lee, Y. Honda, S. E. Kim, C. He, H. Kan, R. Chen, Nonlinear exposure-response associations of daytime, nighttime, and day-night compound heatwaves with mortality amid climate change. *Nat. Commun.* **16**, 635 (2025).

30. S. Safieddine, C. Clerbaux, L. Clarisse, S. Whitburn, E. A. B. Eltahir, Present and future land surface and wet bulb temperatures in the Arabian Peninsula. *Environ. Res. Lett.* **17**, 044029 (2022).

31. J. R. Buzan, M. Huber, Moist heat stress on a hotter Earth. *Annu. Rev. Earth Planet. Sci.* **48**, 623–655 (2020).

32. C. Schwingshackl, J. Sillmann, A. M. Vicedo-Cabrera, M. Sandstad, K. Aunan, Heat stress indicators in CMIP6: Estimating future trends and exceedances of impact-relevant thresholds. *Earth's Future* **9**, e2020EF001885 (2021).

33. P. Wang, L. R. Leung, J. Lu, F. Song, J. Tang, Extreme wet-bulb temperatures in China: The significant role of moisture. *J. Geophys. Res. Atmos.* **124**, 11944–11960 (2019).

34. S. AlSarmi, R. Washington, Recent observed climate change over the Arabian Peninsula. *J. Geophys. Res.* **116**, D11109 (2011).

35. X. Zhang, E. Aguilar, S. Sensoy, H. Melkonyan, U. Tagiyeva, N. Ahmed, N. Kutaladze, F. Rahimzadeh, A. Taghipour, T. H. Hantosh, P. Albert, M. Semawi, M. K. Ali, M. H. Said Al-Shabibi, Z. Al-Oulan, T. Zatari, Imad Al Dean Khelet, S. Hamoud, R. Sagir, M. Demircan, M. Eken, M. Adiguzel, L. Alexander, T. C. Peterson, T. Wallis, Trends in Middle East climate extreme indices from 1950 to 2003. *J. Geophys. Res.* **110**, D22104 (2005).

36. M. Mohamed, A. Othman, A. Z. Abotalib, A. Majrashi, Urban heat island effects on megacities in desert environments using spatial network analysis and remote sensing data: A case study from western Saudi Arabia. *Remote Sens.* **13**, 1941 (2021).

37. C. Hohenegger, P. Korn, L. Linardakis, R. Redler, R. Schnur, P. Adamidis, J. Bao, S. Bastin, M. Behravesh, M. Bergemann, J. Biercamp, H. Bockelmann, R. Brokopf, N. Brüggemann, L. Casaroli, F. Chegini, G. Datseris, M. Esch, G. George, M. Giorgetta, O. Gutjahr, H. Haak, M. Hanke, T. Ilyina, T. Jahns, J. Jungclaus, M. Kern, D. Klocke, L. Kluft, T. Kölling, L. Kornblueh, S. Kosukhin, C. Kroll, J. Lee, T. Mauritsen, C. Mehlmann, T. Mieslinger, A. K. Naumann, L. Paccini, A. Peinado, D. S. Praturi, D. Putrasahan, S. Rast, T. Riddick, N. Roeber, H. Schmidt, U. Schulzweida, F. Schütte, H. Segura, R. Shevchenko, V. Singh, M. Specht, C. C. Stephan, J.S. von Storch, R. Vogel, C. Wengel, M. Winkler, F. Ziemen, J. Marotzke, B. Stevens, ICON-Sapphire: Simulating the components of the Earth system and their interactions at kilometer and subkilometer scales. *Geosci. Model Dev.* **16**, 779–811 (2023).

38. P. Scholz, D. Sidorenko, O. Gurses, S. Danilov, N. Koldunov, Q. Wang, D. Sein, M. Smolentseva, N. Rakowsky, T. Jung, Assessment of the Finite-volumE Sea ice-Ocean Model

(FESOM2.0)—Part 1: Description of selected key model elements and comparison to its predecessor version. *Geosci. Model Dev.* **12**, 4875–4899 (2019).

39. S. Danilov, D. Sidorenko, Q. Wang, T. Jung, The Finite-volumE Sea ice-Ocean Model (FESOM2). *Geosci. Model Dev.* **10**, 765–789 (2017).

40. T. Rackow, X. Pedruzo-Bagazgoitia, T. Becker, S. Milinski, I. Sandu, R. Aguridan, P. Bechtold, S. Beyer, J. Bidlot, S. Boussetta, W. Deconinck, M. Diamantakis, P. Dueben, E. Dutra, R. Forbes, R. Ghosh, H. F. Goessling, I. Hadade, J. Hegewald, T. Jung, S. Keeley, L. Kluft, N. Koldunov, A. Koldunov, T. Kölling, J. Kousal, C. Kühnlein, P. Maciel, K. Mogensen, T. Quintino, I. Polichtchouk, B. Reuter, D. Sármány, P. Scholz, D. Sidorenko, J. Streffing, B. Sützl, D. Takasuka, S. Tietsche, M. Valentini, B. Vannière, N. Wedi, L. Zampieri, F. Ziemen, Multi-year simulations at kilometre scale with the integrated forecasting system coupled to FESOM2.5 and NEMOv3.4. *Geosci. Model Dev.* **18**, 33–69 (2025).

41. ECMWF, IFS Documentation CY48R1 - Part IV: Physical Processes (2023), iFS Documentation CY48R1, Part IV; ECMWF; https://doi.org/10.21957/02054f0fbf.

42. B. C. O'Neill, C. Tebaldi, D. P. van Vuuren, V. Eyring, P. Friedlingstein, G. Hurtt, R. Knutti, E. Kriegler, J.F. Lamarque, J. Lowe, G. A. Meehl, R. Moss, K. Riahi, B. M. Sanderson, The scenario model intercomparison project (ScenarioMIP) for CMIP6. *Geosci. Model Dev.* **9**, 3461–3482 (2016).

43. S. Boussetta, G. Balsamo, G. Arduini, E. Dutra, J. McNorton, M. Choulga, A. Agustí-Panareda, A. Beljaars, N. Wedi, J. Munõz-Sabater, P. de Rosnay, I. Sandu, I. Hadade, G. Carver, C. Mazzetti, C. Prudhomme, D. Yamazaki, E. Zsoter, ECLand: The ECMWF land surface modelling system. *Atmos.* **12**, 723 (2021).

44. P. Janssen, *The Interaction of Ocean Waves and Wind* (Cambridge Univ. Press, 2009).

45. G. Zängl, D. Reinert, P. Rípodas, M. Baldauf, The ICON (ICOsahedral Non-hydrostatic) modelling framework of DWD and MPI-M: Description of the non-hydrostatic dynamical core. *Q. J. Roy. Meteorol. Soc.* **141**, 563–579 (2015).

46. C. H. Reick, V. Gayler, D. Goll, S. Hagemann, M. Heidkamp, J. E. M. S. Nabel, T. Raddatz, E. Roeckner, R. Schnur, S. Wilkenskjeld, JSBACH 3—The land component of the MPI Earth System Model: Documentation of version 3.2 (2021), mPI für Meteorologie; https://doi.org/10.17617/2.3279802.

47. J. R. Buzan, K. Oleson, M. Huber, Implementation and comparison of a suite of heat stress metrics within the Community Land Model version 4.5. *Geosci. Model Dev.* **8**, 151–170 (2015).

48. G. Ning, M. Luo, S. Wang, Z. Liu, P. Wang, Y. Yang, Dominant modes of summer wet bulb temperature in China. *Climate Dynam.* **59**, 1473–1488 (2022).

49. R. Stull, Wet-bulb temperature from relative humidity and air temperature. *J. Appl. Meteorol. Climatol.* **50**, 2267–2269 (2011).

50. R. Davies-Jones, An efficient and accurate method for computing the wet-bulb temperature along pseudoadiabats. *Mon. Weather Rev.* **136**, 2764–2785 (2008).

51. S. Yue, C. Y. Wang, Applicability of prewhitening to eliminate the influence of serial correlation on the Mann-Kendall test. *Water Resour. Res.* **38**, 4–1–4–7 (2002).

52. H. Douville, S. Qasmi, A. Ribes, O. Bock, Global warming at near-constant tropospheric relative humidity is supported by observations. *Commun. Earth Environ.* **3**, 1–7 (2022).

53. S. R. Davis, L. J. Pratt, H. Jiang, The Tokar Gap jet: Regional circulation, diurnal variability, and moisture transport based on numerical simulations. *J. Climate* **28**, 5885–5907 (2015).
